# The conformational stability of pro-apoptotic BAX is dictated by discrete residues of the protein core

**DOI:** 10.1038/s41467-021-25200-7

**Published:** 2021-08-13

**Authors:** Noah B. Bloch, Thomas E. Wales, Michelle S. Prew, Hannah R. Levy, John R. Engen, Loren D. Walensky

**Affiliations:** 1 Department of Pediatric Oncology, Dana-Farber Cancer Institute, Boston, MA USA; 2 Linde Program in Cancer Chemical Biology, Dana-Farber Cancer Institute, Boston, MA USA; 3 Department of Chemistry and Chemical Biology, Northeastern University, Boston, MA USA

**Keywords:** Oncogene proteins, Mass spectrometry, Structural biology

## Abstract

BAX is a pro-apoptotic member of the BCL-2 family, which regulates the balance between cellular life and death. During homeostasis, BAX predominantly resides in the cytosol as a latent monomer but, in response to stress, transforms into an oligomeric protein that permeabilizes the mitochondria, leading to apoptosis. Because renegade BAX activation poses a grave risk to the cell, the architecture of BAX must ensure monomeric stability yet enable conformational change upon stress signaling. The specific structural features that afford both stability and dynamic flexibility remain ill-defined and represent a critical control point of BAX regulation. We identify a nexus of interactions involving four residues of the BAX core α5 helix that are individually essential to maintaining the structure and latency of monomeric BAX and are collectively required for dimeric assembly. The dual yet distinct roles of these residues reveals the intricacy of BAX conformational regulation and opportunities for therapeutic modulation.

## Abstract (web summary)

The pro-apoptotic BAX protein is a monomer under homeostatic conditions and, in response to stress, transforms into oligomers that induce apoptosis. Here, the authors characterize structural features of BAX that individually stabilize the monomer while collectively contributing to oligomerization.

## Introduction

BAX is a BCL-2 family protein whose killer function rids the body of damaged or unwanted cells^1^. The constitutive expression of BAX ensures vigilant surveillance of cellular fidelity yet poses a grave risk to cellular survival if BAX is arbitrarily unleashed. Thus, exquisite conformational regulation is an essential feature of BAX physiology^2^. The latent, cytosolic form of BAX is a 21 kDa monomer composed of a core hydrophobic helix (α5) surrounded by 8 additional α-helices^3^. The N- and C-terminal faces of the protein have auto-inhibitory features with the loop between α1 and α2 overlying a trigger site for BAX activation and the C-terminal α9 helix secured within a regulatory groove. Indeed, installing a disulfide tether that locks the α1-α2 loop or C-terminal helix in place effectively prevents BAX activation^4^. A dimeric structure of full-length BAX further showed that the α9 surface of one monomer could engage the N-terminal face of another in a manner that shields the trigger site, resulting in a less activatable species^5^. Upon direct BAX activation at the trigger site by members of the “BH3-only” subclass of BCL-2 proteins, so named because they only bear the conserved BCL-2 homology 3 motif, the α1–α2 loop is displaced, causing allosteric release of both the C-terminal helix for mitochondrial translocation and the BAX BH3 helix for propagation of activation and oligomerization.

NMR analyses of BAX have shown that conformational perturbations triggered by BH3 motifs, such as that of BIM, or small molecule BH3 mimetics, such as BAM7 and BTSA1, are transmitted through the protein’s α5 core^4,6,7^. A small molecule fragment, BIF-44, which binds at the junction of the α3/α4 and α5/α6 hairpins and sensitizes BAX activation by enhancing the conformational flexibility of the α1–α2 region, also operates by an allosteric mechanism that reverberates through the hydrophobic α5 core^8^. In contrast, inhibitory peptides and molecules, such as the BCL-2 BH4 helix and BAI1, directly engage and stabilize portions of the BAX core, and effectively impede the N-terminal activation of BAX^9,10^. Although generally implicated in conformational activation as a conduit for allosteric regulation, the molecular mechanism by which the central BAX α5 helix alternatively restrains or enables BAX activation is unknown.

Once activated, BAX transforms from a cytosolic monomer into a mitochondrial membrane-embedded oligomer that permeabilizes the outer membrane, releasing a series of apoptogenic signaling factors that commit the cell to death. This execution-phase of BAX activation yields oligomeric forms that are, by definition, structurally distinct from the monomer and actuate the destruction of the mitochondrial membrane. A proposed dimeric building block of BAX oligomers, based on the structure of a complex between two BAX α2–α5 truncates, provided an example of such structural reorganization^11^. The inability of full-length BAX to form a dimeric species and induce mitochondrial apoptosis when α5 is disulfide tethered to α6 suggested that separation of α6 from α5 may represent another key step along the BAX activation pathway^11^ and, notably, involves conformational alteration of the monomeric core.

To elucidate the molecular features of α5 that influence the conformational dynamics of BAX from the inside out, we analyzed its amino acid interaction network, which highlighted a striking nexus of interactions involving α5 residues 113–116. Structure-function studies, spanning alanine mutagenesis, biochemical and cellular analyses, and hydrogen-deuterium exchange mass spectrometry, revealed essential roles for each individual residue in preserving the conformational stability of monomeric BAX, yet a collective role of all four residues in stabilizing the proposed dimeric subcomponent of the BAX oligomer. Our findings implicate a strikingly focal region of BAX architecture with dual roles in regulating the monomeric and dimeric states of this critical arbiter of cell death.

## Results

### Network analysis identifies amino acids 113–116 of the BAX α5 core as a nexus of circumferential interaction

**Fig. 1:**
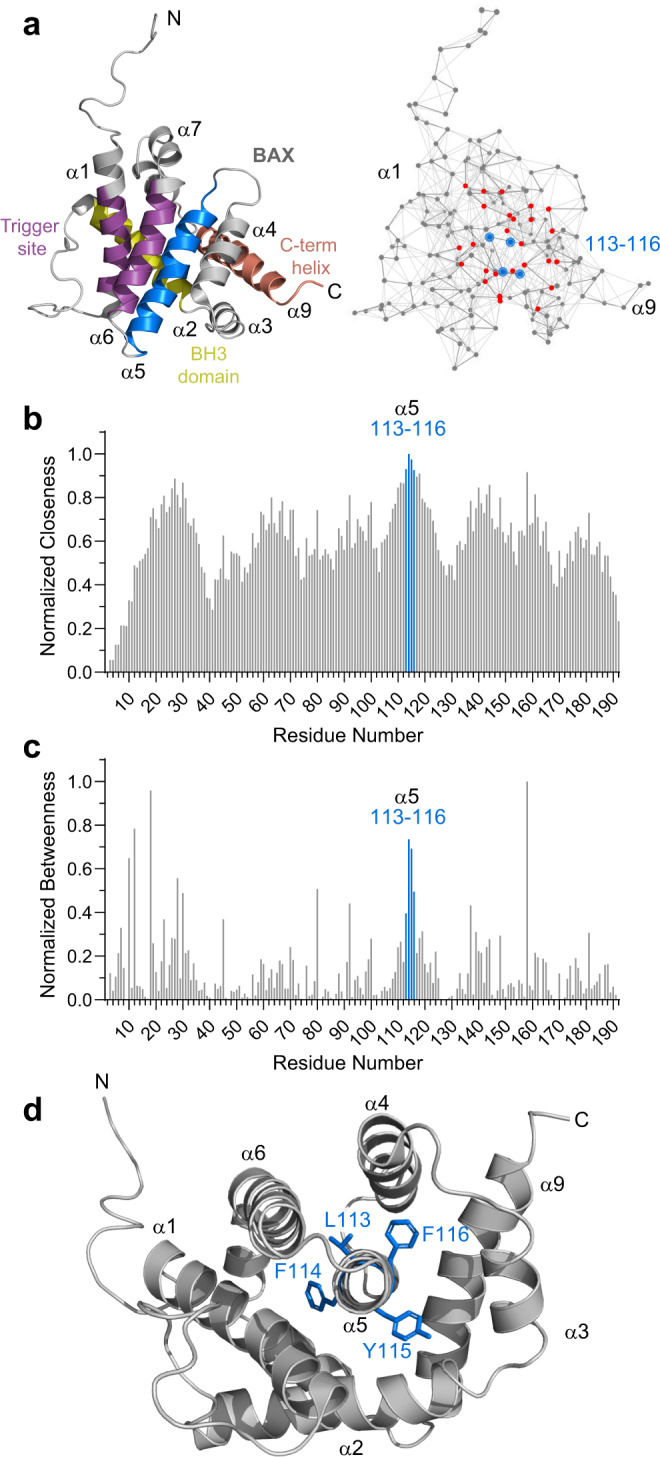
Interaction centrality of core α5 residues 113-116 in the BAX protein network. **a** Structure of monomeric BAX (PDB: 1F16) highlighting regions implicated in BAX activation, including the α1/α6 trigger site (purple), BH3 domain (α2, yellow), C-terminal transmembrane helix (α9, salmon), and the α5 hydrophobic core helix (blue). To the right is a representation of the BAX amino acid interaction network, with residues 113–116 and their network of interacting residues highlighted in blue and red, respectively. **b**, **c** Network centrality analysis of BAX residues plotted as normalized closeness (**b**) and betweenness (**c**). Amino acids 113–116, highlighted in blue, demonstrate the highest closeness values of all BAX residues (**b**) and the highest betweenness values for a consecutive cluster of BAX residues. **d** A view of monomeric BAX down the barrel of α5 demonstrating the radial distribution of residues 113–116 along a single α-helical turn and their collective engagement with every other α-helix of the BAX structure. Source Data are provided as a Source Data File.

To investigate how the allosteric nature of BAX conformational regulation could be mediated by the α5 core, we took an unbiased computational approach based on an analysis of BAX as a network of interactions between each of its amino acid residues (Fig. 1a). Specifically, we used the Network Analysis of Protein Structures (NAPS) algorithm^12^, which has been widely applied to generate hypotheses about how protein structure influences protein function^13–20^. The mathematical parameters of centrality, closeness and betweenness, provide a quantitative framework for the network analysis. Whereas closeness reflects the inverse distance of all shortest paths to a particular residue, betweenness represents the number of shortest bridges incorporating that amino acid. Thus, an amino acid with a high closeness value indicates the network proximity to neighboring residues and that with a high betweenness value signifies a high degree of engagement in shortest path interactions. Strikingly, amino acid F114 of α5 demonstrated the highest closeness value among all BAX residues, and the 113–116 quartet of residues was the highest scoring cluster of consecutive residues for both closeness and betweenness within the BAX network (Fig. 1b, c). Mapping these residues onto the BAX structure revealed that they comprise a complete helical turn of α5 and radiate outward to contact a series of hydrophobic amino acids from every other α-helix in BAX (Fig. 1d, Supplementary Fig. 1). These data implicated α5 residues 113–116 as a potential control point for the conformational regulation of BAX.

### Single alanine mutagenesis of amino acids 113–116 induces ligand-independent BAX activation

**Fig. 2:**
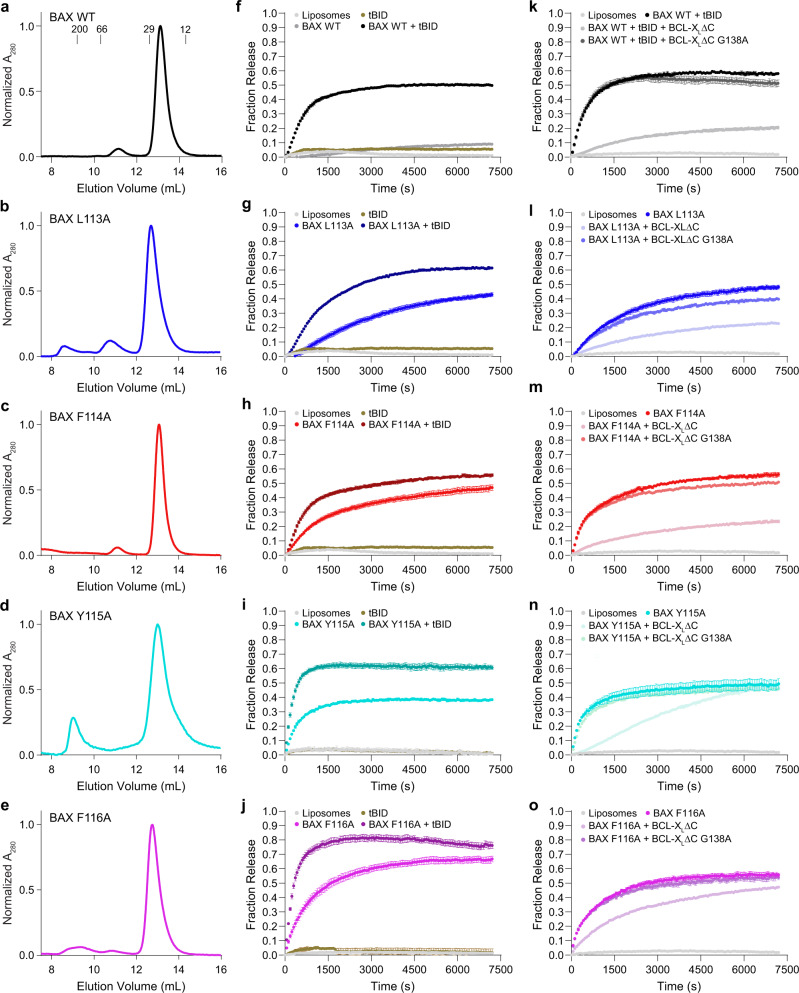
Single alanine mutations of the 113-116 nexus cause BAX autoactivation and membrane poration. **a**–**e** SEC profiles of full-length, recombinant wild-type (**a**), L113A (**b**), F114A (**c**), Y115A (**d**), and F116A (**e**) BAX proteins (5 μM). SEC experiments were repeated twice using independent preparations of protein with similar results. **f**–**j** Liposomal release of ANTS/DPX by wild-type (**f**), L113A (**g**), F114A (**h**), Y115A (**i**), and F116A (**j**) BAX proteins (500 nM) in the presence and absence of the BH3-only activator tBID (10 nM). (**k**) Control experiment documenting the capacity of BCL-X_L_ΔC (10 μM) to inhibit tBID-induced, BAX-mediated liposomal release, whereas G138A point mutagenesis blocks the inhibitory activity of BCL-X_L_ΔC. **l**–**o** Effect of BCL-X_L_ΔC and BCL-X_L_ΔC G138A (10 μM) on the liposomal release autoactivity of L113A (**l**), F114A (**m**), Y115A (**n**), and F116A (**o**) BAX proteins (500 nM). Data are mean ± s.e.m. for liposomal release assays performed in technical quadruplicate and conducted twice using independent preparations of liposomes and proteins with similar results. Source Data are provided as a Source Data File.

To assess the influence of amino acids 113–116 on the conformational stability and functional activity of BAX, we generated recombinant full-length BAX proteins bearing L113A, F114A, Y115A, or F116A single point mutants (Supplementary Fig. 2). In each case, the predominant species generated in solution, as detected by size exclusion chromatography (SEC), was monomeric BAX protein (Fig. 2a–e). We then incubated each BAX construct with liposomes loaded with the fluorophore/quencher pair ANTS/DPX and monitored membrane permeabilization, as quantitated by time-dependent liposomal release of fluorophore, in the presence and absence of the direct BAX activator BH3-only protein, tBID^21,22^. Whereas incubation of liposomes with wild-type BAX had little to no membrane permeabilization effect, robust time-dependent release was observed upon tBID treatment, consistent with direct BH3-triggered BAX activation and functional BAX poration (Fig. 2f). In contrast, and in each case, single alanine mutagenesis of residues 113, 114, 115, or 116 resulted in BAX-mediated liposomal release in the absence of a BH3 ligand, with BAX F116A showing the highest level of autoactivation in the presence of a membrane environment (Fig. 2g–j). Addition of tBID enhanced activation of the BAX mutants even further, indicating that the direct, BH3-triggering mechanism was still intact (Fig. 2g–j). For each mutant, and especially F116A, tBID treatment resulted in a maximal level of liposomal release exceeding that of the wild-type BAX protein, with enhanced release kinetics especially evident for the Y115A and F116A mutants (Fig. 2f–j). We confirmed that alanine mutagenesis had no disruptive effect on liposomal membrane engagement as evidenced by equivalent tBID-induced BAX translocation (Supplementary Fig. 3).

To further confirm that the BAX alanine mutants were operating via the canonical conformational-activation pathway^4^, we tested the capacity of anti-apoptotic BCL-X_L_ΔC to impair BAX activity. We first demonstrated that our recombinant BCL-X_L_ΔC construct was functional and that its inhibitory activity was specifically dependent upon an intact canonical pocket, which is required to capture and neutralize pro-apoptotic BH3 domains^23^. Our positive control experiments showed that tBID-triggered activation of wild-type BAX was effectively suppressed by BCL-X_L_ΔC and that G138A mutagenesis within the BCL-X_L_ΔC pocket abrogated its neutralizing activity (Fig. 2k). For each BAX mutant, co-incubation with BCL-X_L_ΔC impaired autoactivation, whereas BCL-X_L_ΔC G138A had no such effect (Fig. 2l–o). Whereas the efficacy of BCL-X_L_ΔC inhibition of the L113A and F114A mutants was similar to that observed for suppression of tBID-triggered wild-type BAX (Fig. 2k–m), the autoactivity of the BAX Y115A and F116A variants was only partially blocked (Fig. 2n, o), suggesting that the latter mutants are more active, as also evidenced by the shorter time to reach maximal liposomal release in the presence and absence of tBID (2i-j). To confirm that incomplete inhibition of BAX Y115A and F116A was due to a kinetics disadvantage of BCL-X_L_ΔC, we subjected the most active variant, BAX F116A, to incubation with full-length BCL-X_L_, which bears the C-terminal membrane-insertion helix. In contrast to BCL-X_L_ΔC, full-length BCL-X_L_ is independently capable of partitioning to liposomal membranes (Supplementary Fig. 4a) and is indeed more effective at suppressing release by BAX F116A autoactivation in the presence of liposomes (Fig. 2o, Supplementary Fig. 4b). Taken together, these data reveal that just a single point mutation within the 113-116 amino acid nexus of the BAX α5 core causes autoactivation in the presence of liposomal membranes. The observed activity can be enhanced by canonical BH3-triggering and suppressed by canonical anti-apoptotic BH3-in-groove capture, consistent with an on-pathway mechanism of action.

### Functional consequences of individual BAX 113-116 mutagenesis in mitochondria and cells

**Fig. 3:**
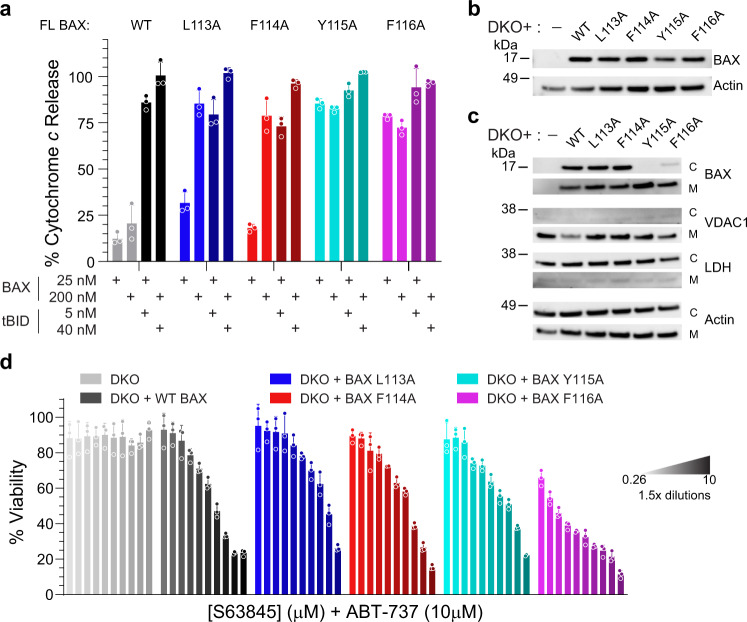
Effect of BAX 113-116 alanine mutagenesis on mitochondrial apoptosis, intracellular localization, and cell viability. **a** Cytochrome *c* release from liver mitochondria purified from *Alb-cre*^pos^*Bax*^f/f^*Bak*^−/−^ mice. Mitochondria were treated with the indicated concentrations of full-length (FL) BAX proteins in the presence or absence of tBID, and cytochrome *c* released into the supernatant was measured by ELISA. Data are mean ± s.d. for cytochrome *c* release assays performed in technical triplicate and conducted twice using independent preparations of proteins and mitochondria with similar results. **b** Western analysis of lysates from *Bax*^−/−^*Bak*^−/−^ MEFs reconstituted with wild-type BAX or the indicated alanine mutants, using BAX and actin antibodies. The analysis was performed twice using independent preparations of proteins with similar results. **c** Western analysis of cytosolic (C) and mitochondrial (M) fractions from *Bax*^−/−^*Bak*^−/−^ MEFs with wild-type BAX or the indicated alanine mutants, using BAX, VDAC1, LDH and actin antibodies. The experiment was performed twice using independent cell cultures and fractionation with similar results. **d** Cell viability of *Bax*^−/−^*Bak*^−/−^ MEFs reconstituted with wild-type BAX or the indicated alanine mutants and co-treated with ABT-737 (10 μM) and the indicated doses of S63845 for 24 h, as measured by CellTiter-Glo. Data are mean ± s.d. for viability experiments performed in technical triplicate and conducted twice using independent cell cultures and treatments with similar results. Source data are provided as a Source Data File.

To evaluate our findings in a physiologic context, we first examined the influence of each single alanine mutant on mitochondrial outer membrane permeabilization, as assessed by BAX-mediated cytochrome *c* release from liver mitochondria isolated and purified from *Alb-cre*^pos^*Bax*^f/f^*Bak*^−/−^ mice^4^. In contrast to the reductionist liposomal release assay, which contains only the core components for biochemical analysis, namely membrane, BAX, a BH3 trigger, and an anti-apoptotic protein, isolated mitochondria contain the full spectrum of proteins and lipids of the native organelle, adding complexity to the experimental system. Consistent with the liposomal release data (Fig. 2g–j), mitochondrial treatment with the individual BAX alanine mutants induced cytochrome *c* release in the absence of a BH3 stimulus (Fig. 3a). Again, the BAX Y115A and F116A variants were the most active, and in the mitochondrial context equally active, inducing near maximal cytochrome *c* release at 25 nM dosing, whereas the BAX L113A and F114A proteins achieved a similar level of activity at 200 nM dosing (Fig. 3a). Wild-type BAX exhibited little to no autoactivation at either protein dose and required triggering with tBID to induce cytochrome *c* release. tBID enhanced BAX-mediated cytochrome *c* release for the alanine mutants as well and was most evident for the relatively less autoactive L113A and F114A variants, particularly at the 25 nM dosing level. All BAX variants achieved complete cytochrome *c* release at the maximal tBID (40 nM) and BAX (200 nM) dosing applied, again indicating that the predominant impact of mutagenesis involved initiation of BAX activation rather than execution.

To assess the impact of alanine mutagenesis of the BAX 113-116 residues in a cellular context, we reconstituted *Bax*^−/−^*Bak*^−/−^ mouse embryonic fibroblasts (MEFs) with wild-type BAX or the individual mutants, selecting for live cells that expressed equivalent levels of BAX protein (Fig. 3b). In comparison to the isolated mitochondrial experiments, the cellular context adds even further complexity to the analysis given the presence of the full spectrum of regulatory BCL-2 family and other proteins within the cytosol and cellular organelles. We first examined how the BAX proteins partitioned between cytosolic and mitochondrial fractions under baseline conditions, in the absence of a stress stimulus. Intriguingly, BAX L113A and BAX F114A proteins were identified in relatively equal proportions between the two fractions, whereas BAX Y115A and F116A were almost exclusively found in the mitochondrial fraction, consistent with an increased propensity for mitochondrial translocation—a feature of autoactivation (Fig. 3c). To measure comparative pro-apoptotic activity of the reconstituted BAX proteins, we treated the individual cell lines with small molecules that selectively target a discrete spectrum of anti-apoptotic proteins, blocking MCL-1 with S63845^24^ and BCL-2/BCL-X_L_ with ABT-737^25^, thereby derepressing native BH3-only protein activators of BAX and disrupting inhibitory interactions between the targeted anti-apoptotic proteins and reconstituted BAX. As a measure of “end stage” BAX activity, cell viability was impaired across all cell lines except for the *Bax*^−/−^*Bak*^−/−^ MEFs, validating that each reconstituted BAX protein—irrespective of BAX 113–116 point mutagenesis that individually influence the afferent step of conformational activation of monomeric BAX — can effectively execute its pro-apoptotic function (Fig. 3d). Although BAX Y115A and F116A were equally hyperactive in inducing cytochrome *c* release from isolated mitochondria and showed similar mitochondrial auto-translocation in cells, MEFs reconstituted with BAX F116A were the most sensitive to anti-apoptotic inhibition (Fig. 3d), consistent with this particular mutant having the fastest kinetics and highest absolute level of liposomal release activity in response to BH3-triggering (Fig. 2j).

### Each residue of the 113–116 nexus plays a regiospecific role in stabilizing the structure of monomeric BAX

**Fig. 4:**
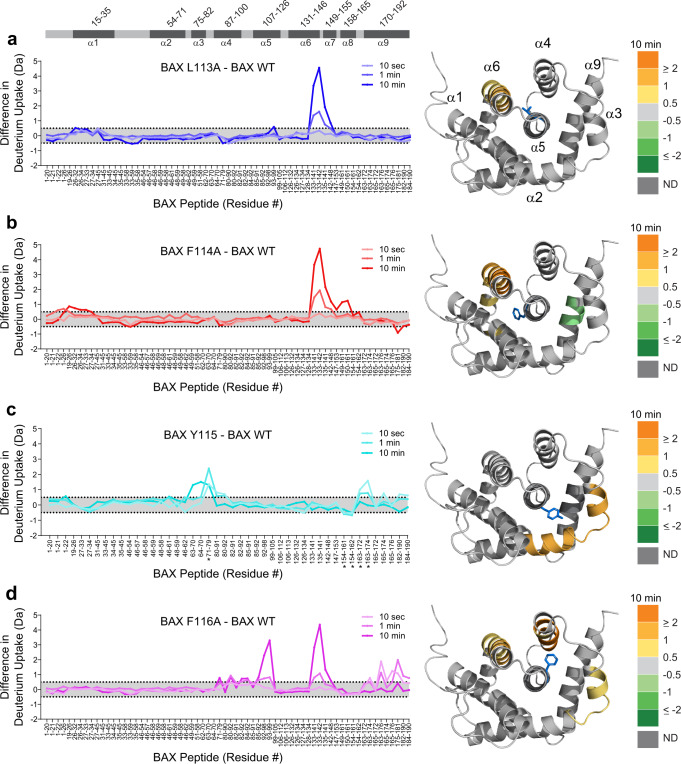
HDX MS reveals regiospecific changes in conformational dynamics upon single alanine mutagenesis of BAX 113-116. **a**–**d** Deuterium difference plots showing the relative deuterium incorporation of BAX L113A (**a**), F114A (**b**), Y115A (**c**), and F116A (**d**) minus the relative deuterium incorporation of BAX wild-type, as measured at 10 sec, 1 min, and 10 min of deuterium labeling. Regions of deprotection (orange) and protection (green) above the 0.5 Da significance threshold at 10 min labeling are mapped onto the solution structure of BAX (PDB: 1F16), with the respective mutated residue colored in blue. HDX MS experiments were performed at least twice using independent preparations of BAX proteins (Supplementary Data 1). The asterisk in (**c**) marks 5 of 315 peptides in the study (63 unique peptides were followed per BAX protein) that display minor EX1 kinetic signatures (bimodal exchange patterns reflective of correlated/cooperative unfolding) at only the earliest time point; all other peptides display EX2 kinetics (uncorrelated local dynamics producing a binomial exchange profile). The HDX MS data used to create this figure can be found in Supplementary Data 2.

To investigate the structural basis for the biochemical and cellular phenotype upon single alanine mutagenesis of the 113-116 nexus, we turned to hydrogen-deuterium exchange mass spectrometry (HDX MS) analysis, which can be used to assess conformational dynamics^26^. HDX MS measures deuterium incorporation into backbone amide hydrogens. Backbone hydrogens in flexible and/or exposed regions of the protein will rapidly exchange with deuterium when the sample is diluted into deuterium buffer. In contrast, backbone amide hydrogens show slowed or suppressed deuterium exchange when they are located in buried domains, in regions where they are involved in hydrogen bonding (such as in α-helices), or in areas engaged in ligand interactions. Here, we compared the HDX MS profile of full-length recombinant BAX in solution with that of the BAX L113A, F114A, Y115A, and F116A single point mutants. In each case, we observed striking, regiospecific consequences of replacing the bulky hydrophobic residue with alanine. Specifically, the L113A mutant showed progressive deprotection over time of α6, which is directly engaged by L113 and implicated in both the mechanism of BH3 triggering^27^ and membrane interaction by its conserved arginine residues^28^ (Fig. 4a). For F114A, the location of increased deuterium exchange shifts to portions of α1, α6 and α7, which are in direct contact with F114, in addition to allosteric protection of the very portion of α9 located at the opposite surface of α5 relative to F114 (Fig. 4b). Y115A mutagenesis caused deprotection of the distal half of α2 and the entirety of α3, to which Y115 points (Fig. 4c). In contrast to L113A and F114A, Y115A mutagenesis induced rapid and transient deprotection of the C-terminal α9 helix, as evidenced by increased deuterium exchange at the earliest labeling time point (Fig. 4c, Supplementary Fig. 5a). F116A mutagenesis also exhibited early deprotection of α9, in addition to progressive deprotection over time of the most distal residue of α2, in addition to α3, α4 and α6 (Fig. 4d, Supplementary Fig. 5b). Among the four constructs, F116A mutagenesis induced the greatest conformational loosening of the BAX structure, as demonstrated by increased deuterium exchange across portions of helices α2, α3, α4, and α6 (Fig. 4d).

These HDX MS data provide a series of mechanistic insights into the relative roles of residues 113-116 in both girding the monomeric structure of BAX and regulating its conformational dynamics. First, the sequential radial deprotection of BAX α-helices that directly face each of the four core α5 residues underscores the critical infrastructural role these residues play in stabilizing monomeric BAX (Fig. 4a–d). Second, the differential regions of deprotection among the four mutants provide a structural explanation for the degree of autoactivation observed in membrane release assays and the differences in mitochondrial partitioning upon reconstitution of the BAX mutants in cells. For example, BAX Y115A and F116A, which were observed to have heightened kinetics of autoactivation in liposomal release assays (Fig. 2i, j) and a lower dosing threshold for autoinduction of mitochondrial cytochrome *c* release (Fig. 3a) are the two mutants that show (1) similar deprotection of the distal portion of α2, the α2-α3 hinge, and α3 (Fig. 4c, d), the very region of BAX containing the critical BH3 helix that is essential to the propagation of BAX activation and oligomerization^29^ and (2) early exposure of α9, whose release from the canonical binding pocket drives mitochondrial translocation^3,4^. Indeed, comparative anti-BAX BH3 pull-down of the four alanine mutants demonstrated selective immunoprecipitation of Y115A and F116A (Supplementary Fig. 6), corroborating the HDX MS results that revealed conformational deprotection of the α2-α3 region. Of the four mutants, F116A mutagenesis results in the fastest kinetics and highest absolute level of liposomal release activity, both alone and in response to a BH3 stimulus (Fig. 2j). Upon targeted inhibition of anti-apoptotic proteins in *Bax*^−/−^*Bak*^*-/-*^ MEFs reconstituted with the BAX mutants, F116A mutagenesis also has the most detrimental effect on cell viability (Fig. 3d). Consistent with these functional data, BAX F116A is the mutant that shows deprotection across the most α-helices by HDX MS (Fig. 4d). Finally, the two mutants with relatively lower yet clearly evident propensity for autoactivation, L113A and F114A, share the common feature of α6 deprotection (Fig. 4a, b), which is also observed for F116A (Fig. 4d). As both a component of the BH3 trigger site and harboring critical arginine residues involved in mitochondrial membrane interaction and poration, α6 has important and separable functions in the initiation and execution phases of BAX activation^28^. Taken together, the HDX MS analysis reveals how the nexus of BAX 113-116 interactions at the α5 core stabilize the monomeric state and further demonstrates how disruption of these interactions, whether by alanine mutagenesis, direct BH3 triggering^4,27^, or pharmacologic modulation^6–8^, can facilitate the transformation of latent BAX into a membrane-disruptive oligomer.

### Distinguishing roles for BAX 113-116 in monomeric versus oligomeric stability

**Fig. 5:**
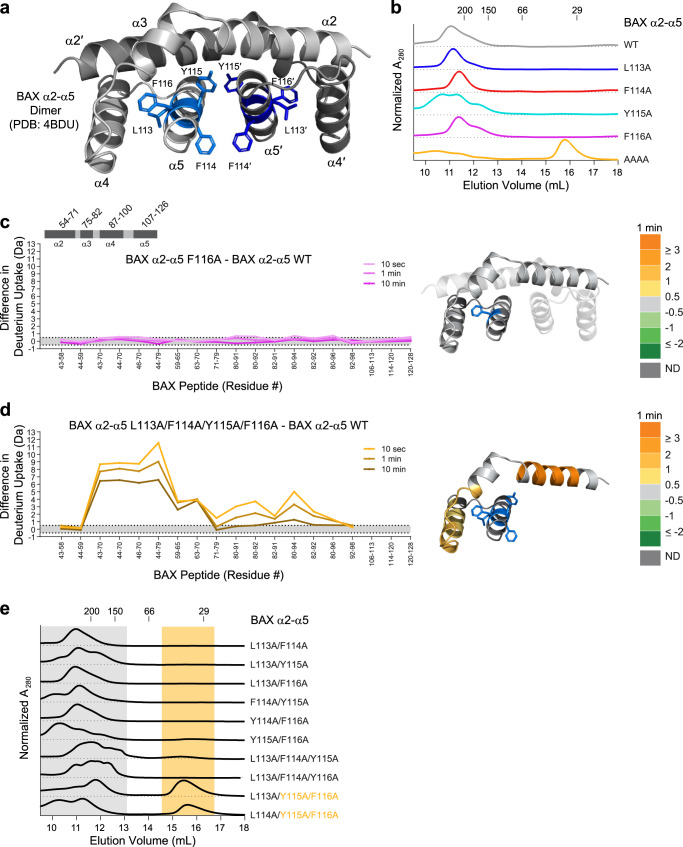
Collective rather than singular mutagenesis of the BAX 113-116 nexus is required to disrupt the conformational stability of the BAX α2–α5 dimer. **a** Crystal structure of dimeric GFP-BAX α2–α5 (PDB: 4BDU) with the N-terminal GFP-tag removed for clarity. The positions of residues 113-116 are shown in blue. **b** SEC of the indicated wild-type and single alanine and quadruple alanine (AAAA) mutant forms of GFP-BAX α2–α5. The dimeric complex of GFP-BAX α2–α5 is observed as a dimer/oligomer of dimers (e.g. tetramerization mediated by GFP). The experiment was performed twice using independent preparations of proteins with similar results. (**c**–**d**) Deuterium difference plots showing the relative deuterium incorporation of (**c**) BAX α2–α5 F116A and (**d**) BAX α2–α5 L113A/F114A/Y115A/F116A minus the relative deuterium incorporation of wild-type BAX α2–α5, as measured at 10 sec, 1 min, and 10 min of deuterium labeling. Regions of deprotection (orange) and protection (green) above the 0.5 Da significance threshold at 1 min labeling are mapped onto the crystal structure (PDB: 4BDU) of the BAX α2–α5 dimer for (**c**) and a monomeric subunit for (**d**), with the respective mutated residue(s) colored in blue. HDX MS experiments were performed at least twice using independent preparations of BAX proteins. All peptides display EX2 kinetics (uncorrelated local dynamics producing a binomial exchange profile). The HDX MS data used to create this figure can be found in Supplementary Data 2. **e** SEC profiles of the indicated double and triple alanine mutant forms of GFP-BAX α2–α5. Elution positions of wild-type dimer and quadruple-mutant monomer are shaded in grey and gold, respectively, as references. The experiment was performed twice using independent preparations of proteins with similar results. Source data are provided as a Source Data file.

Intriguingly, a prior cellular study showed that quadruple mutagenesis of BAX 113-116 in the context of the full-length protein abrogated the pro-apoptotic activity of BAX, consistent with a defect in the execution phase of BAX activation, and homo-oligomerization in particular^30^. A dimeric species of BAX α2–α5 was proposed to function as an essential subunit of homo-oligomerization^30^ and a crystal structure of the dimeric truncate (GFP-BAX α2–α5) was subsequently solved^11^. Most recently, NMR analysis of this dimeric species (tagless) in bicelles delineated portions of BAX α2–α5 that can directly engage membrane lipids^31^. The specific amino acid interactions of BAX 113–116 are distinct in the latent, full-length monomer and the dimeric BAX α2–α5 complex (Figs. 1d, 5a). To dissect the specific roles of BAX residues 113–116 in monomeric versus dimeric stability, we generated BAX α2–α5 truncates bearing the individual alanine mutants and the quadruple mutant, and evaluated dimeric assembly by SEC. We strikingly found that the individual alanine mutants had no disruptive effect on dimeric assembly (observed as a GFP-mediated dimer/oligomer of BAX α2–α5 dimers^11^), whereas quadruple mutagenesis completely disrupted formation of the complex (Fig. 5b), as observed in the context of full-length BAX in cells^30^. To examine and compare the conformational consequences of individual versus collective alanine mutagenesis in the dimeric context, we performed HDX MS analyses of GFP-BAX α2–α5 and its F116A and quadruple alanine mutants. Whereas single F116A mutagenesis induced the most dramatic conformational consequences in monomeric full-length BAX, no effect was observed in the context of the BAX α2–α5 dimer (Fig. 5c), consistent with the SEC analysis (Fig. 5b). Instead, quadruple mutagenesis induced marked conformational deprotection in BAX α2–α5 (Fig. 5d), consistent with monomeric exposure of previously buried motifs due to blockade of dimer formation.

Finally, to discern the relative contributions of each residue of the 113–116 nexus to formation of the core BAX α2–α5 dimer, we undertook a comprehensive mutational analysis, generating the 6 combinations of double mutants and four combinations of triple mutants (Fig. 5e). Of note, the BAX α2–α5 system was especially useful for this comparative analysis in solution owing to the inability to generate the quadruple mutant construct in full-length form. Like the individual alanine mutants (Fig. 5b), all variants bearing double alanine mutations maintained the capacity to self-assemble. The triple BAX α2–α5 mutants L113A/F114A/Y115A and L113A/F114A/F116A also eluted as a complex, indicating that retention of native residues Y115 or F116 is sufficient to preserve dimerization and thus rescue quadruple alanine mutagenesis. Interestingly, when Y115 and F116 are mutated in the context of a triple alanine mutation, specifically L113A/Y115A/F116A and F114A/Y115A/F116A, dimerization is partially impaired, as demonstrated by the presence of a monomeric species (Fig. 5e). These data highlight (1) key combinatorial roles of residues Y115 and F116 in BAX self-association; (2) the capacity of L113 or F114 to maintain at least partial self-association of the triple mutant bearing Y115A and F116A mutations; and (3) L113 and F114 in combination can completely overcome Y115A/F116A mutagenesis in the context of the double mutant. These findings reinforce the conclusion that each of the 113–116 residues can independently influence the stability of monomeric BAX, but this nexus of residues functions collectively to regulate the formation and stability of the core dimeric species of BAX α2–α5. Indeed, comparative network analysis demonstrates that in the dimeric assembly, a series of residues in the α2 helix and proximal α3 have substantially increased closeness and betweenness values, reflecting additional reinforcement of the dimeric assembly compared to monomeric BAX (Supplementary Fig. 7, 8). Correspondingly, we observe that dimeric assembly persists despite single alanine mutagenesis of residues 113–116 and instead quadruple mutagenesis is required for complete complex disruption, highlighting the distinct roles of these key residues and their interactions (Figs. 1d, 5a, Supplementary Fig. 1, 7, 8) in regulating the conformational dynamics of monomeric versus oligomeric BAX.

## Discussion

BAX is a relatively small protein of only 21 kDa in size yet has the capacity to transform into a higher order species that is responsible for cellular self-destruction. Given its lethal potential, BAX is subject to a remarkable degree of regulation. Such physiologic mechanisms include auto-inhibitory interactions at the N- and C-terminal faces of BAX^3,4^ and targeted inhibition by anti-apoptotic members of the BCL-2 family^1,9,23^ and other proteins, such as the cytomegalovirus vMIA protein^32^. BAX activation can occur directly, through triggering interactions between select BH3-only proteins and a binding site formed by the confluence of α1 and α6 residues at the N-terminal face^27^, and indirectly, by BH3-only protein displacement of activated BAX from neutralizing heterodimeric complexes with anti-apoptotic proteins^33,34^. BAX can also be activated by heat^35^, highlighting that a discrete energetic threshold exists that, when breached, can transform an otherwise stabilized architecture into a volatile one. In contrast to heat-mediated protein denaturation and loss-of-function, enhancing the structural flexibility of BAX results in a dramatic conformational change that underlies a gain-of-function, namely higher order oligomerization and permeabilization of the mitochondria.

A series of structure-function studies have demonstrated the critical role of allostery in mediating the conformational regulation of BAX^3,4,36,37^. For example, biochemical and structural analyses using full-length recombinant BAX protein revealed that BH3 ligands capable of displacing the α1-α2 loop from the N-terminal surface, can initiate BAX activation by allosteric release of α9^4^. In contrast, synthetic tethering of the α1-α2 loop or α9 to its respective N- and C-terminal grooves effectively restrains monomeric BAX^4^. The discovery and structure-function analyses of additional BAX-interacting ligands have further reinforced the allostery theme of BAX regulation. A small molecule that binds to the confluence of the α3/α4 and α5/α6 hairpins sensitizes BAX activation by allosteric mobilization of the α1-α2 loop and α2 at the opposite side of the protein^8^. Non-enzymatic covalent derivatization of C126 by the mitochondrial lipid electrophile, 2-hexadecenal, induces allosteric chemical shift perturbations at both the α1-α2 loop and α9, as observed by NMR analysis, and enhances BAX activity^38^. In contrast, inhibitors such as the BCL-2 BH4 domain, which engages BAX at a groove formed by the confluence of the α2/α3 and α5/α6 hairpins, allosterically restrain α1 and the α1-α2 loop, and thereby impair BAX activation^9^. Just recently, eltrombopag, a structural analog of the BAX-activating molecules BAM7^6^ and BTSA1^7^, was shown to engage the α1/α6 trigger site yet inhibit rather than activate BAX by stabilizing key regions of the BAX architecture, including the α1-α2 loop and α9^39^. All of these studies focus on how “external forces” drive the conformational dynamics of BAX, but how are these mechanisms mediated from within?

Here, we find that a nexus of hydrophobic residues that radiate outward from the central portion of the α5 core provides critical stabilizing interactions to monomeric BAX. Mutating any single residue of the 113–116 region leads to heightened BAX autoactivation in the membrane environment, with the two most active mutations, Y115A and F116A, mobilizing both α2 and α9, two domains especially critical to BAX function. By nature of the very location of the four residues and their interactions with each surrounding BAX helix, natural protein ligands that engage their respective binding sites on the BAX surface can directly influence, positively or negatively, the stability of this critical network and thus the threshold for conformational activation. Correspondingly, peptide^9,21,32,39^ and small molecule^6,7,10,39^ mimetics of these interacting motifs can disrupt or reinforce this nexus for potential therapeutic benefit in diseases of pathologic cellular excess or deficiency.

Another unique feature of the BAX 113–116 nexus is that it subserves two distinct roles along the BAX activation pathway. In addition to having singular and independent roles in stabilizing monomeric BAX, amino acids 113–116 function collectively, rather than individually, to stabilize a dimeric conformation of BAX that may represent a key subunit of higher order oligomers. Distinct and step-specific roles of discrete residues along the BAX activation continuum has emerged as a remarkable feature of this pro-apoptotic mechanism. For example, we recently demonstrated that mutagenesis of the α6 surface residue I133 disrupted BH3 binding to the trigger site and thus the initiation of BAX activation but had no adverse effect on the execution phase steps of mitochondrial translocation, self-association, and membrane poration^28^. In contrast, mutagenesis of R134, a surface residue immediately adjacent to I133 and also a component of the N-terminal trigger site had no effect on BH3-mediated initiation of BAX activation and instead impaired membrane tropism and oligomeric poration^28^. Here we find that, depending on the stage-specific structural context of monomer vs. dimer, BAX α5 core residues 113–116 and their network of hydrophobic interactions either have individual or cumulative functional importance.

Protein hydrophobic cores can often play a key role in protein assembly and allosteric function. For example, protein kinases rely on allosteric switches to confer on-off enzymatic function. Their prototypical architecture involves two core hydrophobic spines that mediate signal transduction between distal sites^40^. Disruption of these central nodes of communication by point mutagenesis typically leads to loss of enzymatic function^40,41^. In contrast, loosening of the hydrophobic interactions within the core of monomeric BAX by point mutagenesis activates protein function in the membrane environment, leading to oligomeric assembly and poration. Transient exposure of hydrophobic BAX surfaces drives this structural transformation, as evidenced by HDX MS studies that revealed initial deprotection of the critical BAX α2 and α9 regions upon BAX activation, followed by prompt protection as oligomerization ensues^9,28,38^. An alternative mechanism for regulated protein oligomerization upon hydrophobic domain exposure is exemplified by gasdermin, whose N-terminal pore-forming monomer is auto-inhibited by a C-terminal region until cleaved by inflammatory caspases, which leads to dissociation of the C-terminal fragment and N-terminal protein self-association and membrane poration^42,43^. Whereas stress conditions, such as inflammation, temperature elevation, or nutrient or oxygen deprivation, typically lead to protein dysfunction, it is fitting that BAX destabilization instead induces protein activation, so that it can execute its mitochondrial permeabilization and cell-killing functions. Indeed, we find that disrupting key interactions of the BAX α5 core is tantamount to “pulling the pin” of the cell’s pro-apoptotic grenade.

## Methods

### Protein network analysis

Network models of BAX were generated using the NAPS server (https://bioinf.iiit.ac.in/NAPS/) as unweighted atom pair contact networks with default parameters. Monomeric BAX was modeled using the NMR solution structure (PDB: 1F16 [10.2210/pdb1F16/pdb]) and dimeric BAX was modeled using the α2–α5 truncate crystal structure (PDB: 4BDU [10.2210/pdb4BDU/pdb], BAX amino acids 54-122). For the monomer-dimer comparison, the output of the network analysis for BAX amino acids 54-122 was analyzed and values normalized using Prism software (Graphpad).

### Recombinant protein expression and purification

Recombinant full-length, wild-type BAX in the pTYB1 vector was expressed in *Escherichia coli* as previously reported^3,8,27^. Point mutations were generated by PCR-based mutagenesis (Q5 Site-Directed Mutagenesis Kit, New England BioLabs) and confirmed by DNA sequencing. Transformed *E. coli* were cultured in LB medium containing carbenicillin (0.1 g/l), grown to an optical density (OD) of 0.6–0.8, and protein expression was induced by the addition of 1.0 mM IPTG at 30 °C for 4 h. Bacterial pellets were resuspended in lysis buffer (20 mM Tris, 250 mM NaCl, pH 7.2) containing protease inhibitor tablets (Roche) and lysed over two passages through a microfluidizer (Microfluidics) on ice. The soluble fraction was isolated by centrifugation at 48,384 x g for 45 min at 4 °C. BAX protein was purified by chitin affinity chromatography using chitin resin (NEB) on a gravity flow column. The intein and affinity tag were cleaved using 10 mg/mL dithiothreitol at 4 °C for 12-36 h. The full-length, tagless protein was eluted, concentrated, and purified by SEC in fast protein liquid chromatography (FPLC) buffer (20 mM HEPES-KOH, 150 mM KCl, pH 7.2) using a Superdex S-75 (GE Healthcare) column on an FPLC system (AKTA Pure, GE Healthcare Life Sciences). Protein purity and identity was confirmed by western analysis using the 2D2 mouse monoclonal BAX antibody (Santa Cruz Biotechnology Cat# sc-20067; RRID: AB_626726; 1:200).

Recombinant full-length BCL-X_L_ (1-233) was cloned into the pTYB1 vector by HiFi DNA assembly (NEBuilder HiFi DNA Assembly Master Mix, NEB). BCL-X_L_ΔC (1-209) and BCL-X_L_ΔC G138A were then generated by PCR and PCR-based mutagenesis (Q5 Site-Directed Mutagenesis Kit, New England BioLabs), respectively, and confirmed by DNA sequencing. Expression and purification were performed as described above for BAX, with expression induced using 0.5 mM IPTG overnight at 16 °C. Protein purity and identity was confirmed by western analysis using a mouse monoclonal anti-BCL-X_L_ (2H12) antibody (Abcam Cat# ab270253; RRID: AB_2536307; 1:1000).

BAX α2–α5 (53–128) truncates containing N-terminal His_6_- and GFP-tags, and bearing C62S and C126S mutations, were cloned into pET28a by HiFi DNA Assembly (NEB). Point mutations were performed by PCR-based mutagenesis (Q5 Site-Directed Mutagenesis Kit, New England BioLabs) and confirmed by DNA sequencing. Transformed *E. coli* were grown and induced as described for BCL-X_L_ except that kanamycin (0.05 g/l) was used in place of carbenicillin. Bacterial pellets were lysed in TBS (20 mM Tris, 150 mM NaCl, pH 8.0). Clarified lysate was applied to Ni-NTA agarose and washed with a gradual imidazole gradient before elution in TBS containing 200 mM imidazole. Eluted N-His_6_-GFP-BAX α2–α5 was dialyzed overnight in TBS lacking imidazole, concentrated, and further purified by SEC using a Superdex S-200 (GE Healthcare) gel filtration system.

The primers used to generate recombinant BAX and BCL-X_L_ constructs are shown in Supplementary Table 1.

### SEC analysis of BAX mutants

Purified monomeric BAX proteins were diluted to 5 μM in 200 μL of FPLC buffer (see above) and incubated at 25 °C for 30 min before subjecting the sample to SEC analysis using a Superdex S-75 column. The data were analyzed using Microsoft Excel 2016 and Prism 8 (Graphpad) software.

### Liposomal release assay

Large unilamellar vesicles (LUVs) encapsulating the fluorophore/quencher pair 8-aminonapthalene-1,3,6-trisulfonic acid (ANTS) and p-xylene-bis-pyridinium bromide (DPX) were formed by liposome extrusion and purified by SEC. Briefly, a lipid mixture that mimics the composition of the mitochondrial outer membrane was generated by dissolving a 48:28:10:10:4 molar ratio of phosphatidylcholine, phosphatidylethanolamine, phosphatidylinositol, dioleoyl phosphatidylserine, and tetraoleolyl cardiolipin (Avanti Polar Lipids) in chloroform. To produce lipid films, nitrogen gas was used to evaporate the chloroform and the lipids were dried under high vacuum overnight. For long-term storage, lipid films were maintained in a nitrogen atmosphere at −80 °C. Lipid films were resuspended in 1 mL of liposomal release assay buffer (20 mM HEPES-KOH, 150 mM KCl, 5 mM MgCl_2_, pH 7.2) containing the fluorophore/quencher pair ANTS (12.5 mM) and DPX (45 mM). Liposomes were formed by exposing the resuspended lipid mixture to five freeze/thaw cycles and then extruding the liposomes using a 100 nm polycarbonate membrane eleven times. Liposomes were then purified from unencapsulated ANTS and DPX using a Sepharose CL-2B size exclusion column (GE Healthcare). The liposomal release assay was performed using an M1000 Infinite (Tecan) equipped with i-control 1.11 software (Tecan). ANTS fluorescence (355 nm excitation, 540 nm emission, 20 nm slit width) was monitored every 60 s after the addition of recombinant BAX proteins (500 nM), with or without tBID (10 nM) or BCL-X_L_ proteins (10 μM). Maximal release was determined by the addition of Triton X-100 to a final concentration of 0.625% (v/v). Fraction release was calculated as ((F-F_0_)/(F_100_-F_0_)), where F is the observed fluorescence at a given time, and F_0_ and F_100_ represent baseline and maximal fluorescence, respectively. The data were analyzed using Microsoft Excel 2016 software and plotted with Prism 8 software (Graphpad).

### Liposomal Translocation Assay

Liposomes (40 μL) were incubated with (1) BAX proteins (1 μM) treated with tBID (200 nM) for 15 min or (2) BCL-X_L_ proteins (1 μM) for 45 min in liposomal release assay buffer at room temperature. Protein-containing solutions were then added to a Sepharose CL-2B (3 mL, GE Healthcare) size exclusion column equilibrated with liposomal release assay buffer and 14 equivalent fractions (250 μL) were collected. Liposome-containing fractions were identified by adding 10% Triton X-100 to the fractions and measuring fluorescence associated with ANTS/DPX release. SDS PAGE followed by western blot analysis was conducted to identify BAX or BCL-X_L_ in the fractions using 2D2 mouse monoclonal BAX antibody (Santa Cruz Biotechnology Cat# sc-20067; RRID: AB_626726; 1:200) or mouse monoclonal anti-BCL-X_L_ (2H12) antibody (Abcam Cat# ab270253; RRID: AB_2536307; 1:1000), respectively.

### Mitochondrial cytochrome *c* release assay

Mitochondria from the livers of *Alb-cre*^pos^*Bax*^f/f^*Bak*^−/−^ mice were isolated and release assays performed as described previously^21^. Briefly, mitochondria (1 mg/mL) were incubated with recombinant BAX proteins (25 or 200 nM) in the presence or absence of tBID (5 or 40 nM, respectively), for 45 min at room temperature in experimental buffer (200 mM mannitol, 70 mM sucrose, 1 mM EDTA, 10 mM HEPES, pH7.4). Control wells contained mitochondria and buffer, with and without added 1% Triton X-100 (v/v). Following incubation, plates were centrifuged for 15 min at 2,055 x g and 50 μL of supernatant was analyzed using the Rat/Mouse Cytochrome c Quantikine ELISA Kit (R&D Systems) according to the manufacturer’s instructions using SoftMax Pro 7.0.2 software. Percentage cytochrome *c* released into the supernatant (%cytochrome *c* release) was calculated according to the following equation: %cytochrome *c* release = [cyto *c*_sup_ − cyto *c*_buffer_]/[cyto *c*_max_ − cyto *c*_buffer_] × 100, where cyto *c*_sup_ − cyto *c*_buffer_ and cyto *c*_max_ − cyto *c*_buffer_ represent the amount of cytochrome *c* specifically released into the supernatant upon treatment with the indicated conditions or 1% (v/v) Triton X-100, respectively. The above animal-derived materials (liver mitochondria) were obtained from *Alb-cre*^pos^*Bax*^f/f^*Bak*^−/−^ mice in accordance with the guidelines and regulations set forth by the Institutional Animal Care and Use Committee of the Dana-Farber Cancer Institute and in compliance with approved study protocol #06-004. Animals were housed in microisolater cages at an ambient temperature of 68–79 °F and humidity of 30–70%, with 12-h light/dark cycles.

### Retroviral transduction and cell culture

Human BAX constructs (wild-type, L113A, F114A, Y115A, and F116A) were cloned (Genewiz) into the pMIG II (pMSCV-IRES-GFP II) vector (Addgene #52107), and the presence of insert and the indicated mutations were confirmed by DNA sequencing. Transfection of the packaging cell line GPG-293 yielded amphotropic retroviral particles, which were collected by filtration and precipitation with PEG-it solution (System Biosciences), and then used to reconstitute *Bax*^−/−^*Bak*^−/−^ MEFs with the indicated BAX mutants, as described^27,44^. The reconstituted MEFs were sorted for GFP-positivity over two rounds of flow cytometry to ensure comparable levels of expression. Transduction was verified by BAX western analysis using the 2D2 antibody (Santa Cruz Biotechnology Cat# sc-20067; RRID: AB_626726; 1:200). Cells were maintained in Dulbecco’s Modified Eagle Medium (GIBCO) with 10% FBS, 100 U/mL penicillin and streptomycin, and 2 mM glutamine. Cells were verified as mycoplasma-negative using the MycoAlert mycoplasma detection kit (Lonza Biologics).

### Mitochondrial isolation from reconstituted MEFs

*Bax*^−/−^*Bak*^−/−^ MEFs and those reconstituted with the indicated BAX proteins were cultured as described above and 2 × 10^7^ cells were harvested for fractionation. Cell pellets were resuspended in isolation kit buffers (ThermoFisher) and lysed by Dounce homogenization. Mitochondrial and cytosolic fractions were purified by differential centrifugation, performed in accordance with the manufacturer’s protocol. Mitochondria were lysed in mitochondrial lysis buffer (25 mM Tris, 150 mM NaCl, 2% CHAPS, pH 7.2) and protein quantitation of the supernatant and cytosolic fractions was performed using the BCA assay (ThermoFisher). Samples were subjected to electrophoresis and western blotting using the 2D2 antibody (Santa Cruz Biotechnology Cat# sc-20067; RRID: AB_626726; 1:200). Western analysis using VDAC1 (Abcam cat #ab186321; 1:1000), LDH (Abcam cat #ab47010, RRID:AB_1952042; 1:1000), and actin (Cell Signaling Technology Cat# 5125, RRID:AB_1903890; 1:1000) antibodies was performed to verify mitochondrial and cytosolic fractions, and equal protein loading, respectively.

### Cell viability assay

*Bax*^−/−^*Bak*^−/−^ MEFs and those reconstituted with the indicated BAX proteins were cultured as described above, plated in 96-well plates (1.5 × 10^3^ cells per well), and allowed to adhere for 24 h. The cells were then treated with 10 μM ABT-737 (Cayman Chemical) and the indicated concentration of S63845 (SelleckChem), or vehicle (0.05% and 0.1% DMSO, respectively) for 24 h. Cell viability was measured by CellTiter-Glo assay (Promega) with luminescence read on a Spectramax M5 microplate reader equipped with SoftMax Pro 7.0.2 software.

### Hydrogen deuterium exchange mass spectrometry

HDX MS analyses of BAX proteins were performed as described previously^9^, with details specific to the experiments conducted here provided in Supplementary Data 1, per the recommended format^45^. Briefly, BAX aliquots were freshly prepared in biological replicates prior to HDX MS analysis with stock concentrations in 20 mM HEPES, 150 mM KCl, 1 mM MgCl_2_, pH 7.2, H_2_O as follows: BAX WT: 22 μM; BAX L113A: 29 or 26 μM; BAX F114A: 29 or 18.6 μM; BAX Y115A: 6.8 μM; BAX F116A: 16 or 24 μM. Deuterium labeling was initiated with an 18-fold dilution into D_2_O buffer (20 mM HEPES, 150 mM KCl, 1 mM MgCl_2_, pD 7.2, 99.9% D_2_O). GFP-tagged BAX α2–α5 proteins were prepared similarly in stock concentrations in TBS (20 mM Tris, 150 mM NaCl) as follows: BAX α2–α5 WT: 20 μM; BAX α2–α5 F116A: 20 μM; BAX α2–α5 L113A/F114A/Y115A/F116A: 20 or 13 μM. Deuterium labeling was initiated with an 18-fold dilution into D_2_O buffer (20 mM Tris, 150 mM NaCl, pD 8.0, 99.9% D_2_O). After each labeling time (10 sec, 1 min, 10 min) at 20 °C, the labeling reaction was quenched with the addition of ice-cold quenching buffer (full-length BAX: 0.8 M guanidinium chloride, 0.8% [v/v] formic acid, pH 2.0, H_2_O; GFP-BAX α2–α5: 4 M guanidium chloride, 200 mM potassium phosphate, pH 2.3, 0.72 M TCEP, H_2_O) and analyzed immediately using a Waters HDX system coupled to a Waters-G2Si mass spectrometer operated in HDMS^E^ mode. Deuterated and control samples were digested online at 15 °C using an AffiPro Nepenthesin-2 column and peptides were trapped and desalted on a VanGuard Pre-Column trap for 3 min at 100 μL/min. Peptides were then eluted from the trap using a 5–35% gradient of acetonitrile over 6 min at a flow rate of 100 μL/min, and separated using an ACQUITY UPLC HSS T3, 1.8 μm, 1.0 mm × 50 mm column with the main cooling chamber held at 0.0 ± 0.1 °C for the entire time of the measurements. Peptides were identified from replicate HDMS^E^ analyses (as detailed in Supplementary Data 1) of undeuterated control samples using PLGS 3.0.1 (Waters Corporation). Peptide masses were identified from searches using non-specific cleavage, no missed cleavages, no PTMs, a minimum of 250 ion counts for low energy threshold, and a 50 ion count threshold for their fragment ions, with no set minimum peptide length. No false discovery rate (FDR) control was performed. The peptides identified in PLGS (excluding all neutral loss and in-source fragmentation identifications) were then analyzed in DynamX 3.0 (Waters Corporation) implementing a minimum products per amino acid cutoff of 0.25, at least two consecutive product ions (see Supplementary Data 1). A database containing only the sequence from human BAX (UniprotKB Q07812) was used with no cleavage specificity and no PTMs considered to refine the peptide list. Those peptides meeting the filtering criteria to this point were further processed by DynamX 3.0 (Waters Corporation). The relative amount of deuterium in each peptide was determined using DynamX 3.0 by subtracting the centroid mass of the undeuterated form of each peptide from the deuterated form, at each time point, for each condition. These deuterium uptake values were used to generate all uptake graphs and difference maps. The error of determining the average deuterium incorporation for each peptide was at or below ±0.25 Da. Deuterium levels were not corrected for back exchange and thus reported as relative^46^.

### Anti-BAX BH3 Immunoprecipitation

BAX proteins (9 μM) were prepared in a 20 μL solution of PBS. After equilibrating for 15 min at room temperature, 10 μL of each sample was added to a 3% BSA in PBS solution (250 μL) containing 15 μL BAX BH3 antibody (Abcepta Cat# AP1302a; RRID:AB_2227716) for 1 hr at 4 °C. Preclarified Pierce Protein A/G agarose beads (50 μL) (ThermoFisher) were then added to the BAX protein/antibody mixture and incubated for an additional 2 hr at 4 °C. The agarose beads were spun down, washed three times with 1 mL of 3% BSA in PBS, resuspended in 50 μL of SDS-sample buffer and boiled for 5 min. Inputs (2%) and immunoprecipitation samples (20 μL) were subjected to SDS PAGE and western blot analysis was conducted using 2D2 mouse monoclonal BAX antibody (Santa Cruz Biotechnology Cat# sc-20067; RRID: AB_626726; 1:200).

### Statistical methods

Prism software (Graphpad) was used for data analysis, and calculating mean, s.d., and s.e.m. values.

### Biological materials

Plasmids and cell lines are available upon request to the corresponding author.

### Reporting summary

Further information on research design is available in the Nature Research Reporting Summary linked to this article.

## Supplementary information

Supplementary Information

Description of Additional Supplementary Files

Supplementary Data 1

Supplementary Data 2

Reporting Summary

## Data Availability

All data generated or analyzed for this study are included in this manuscript and its supplementary information. HDX MS have been deposited to the ProteomeXchange Consortium via the PRIDE^47^ partner repository with the dataset identifier PXD024479. Structures corresponding to PDB 1F16 and 4BDU were used in this study. Source data are provided with this paper.

## Source data

Source Data
