# Supplementary material for: The conformational stability of pro-apoptotic BAX is dictated by discrete residues of the protein core
Source: Nat Commun. 2021 Aug 13;12:4932. doi: 10.1038/s41467-021-25200-7 (PMC8363748; doi:10.1038/s41467-021-25200-7)
Supplement: Supplementary file 1 — Supplementary Information [file 41467_2021_25200_MOESM1_ESM.pdf]

The Conformational Stability of Pro-apoptotic BAX is Dictated by Discrete Residues of the Protein Core

Noah B. Bloch, Thomas E. Wales, Michelle S. Prew, Hannah R. Levy, John R. Engen, and Loren D. Walensky

SUPPLEMENTARY INFORMATION

Supplementary Figures and Figure Legends 1-8

Supplementary Table 1

a

Residue	Interacting Residue	Location
L113	92, 99, 100	α4
	108, 109, 110, 111, 112, 114, 115, 116, 117, 118	α5
	143	α6
	148	α7
F114	27	α1
	63	α2
	110, 111, 112, 113, 115, 116, 117, 118	α5
	144	α6
	158, 161	α8
Y115	30	α1
	67, 70, 71	α2
	80	α3
	111, 112, 113, 114, 116, 117, 118, 119	α5
F116	91, 92, 95, 99	α4
	111, 112, 113, 114, 115, 117, 118, 119, 120	α5
	181	α9

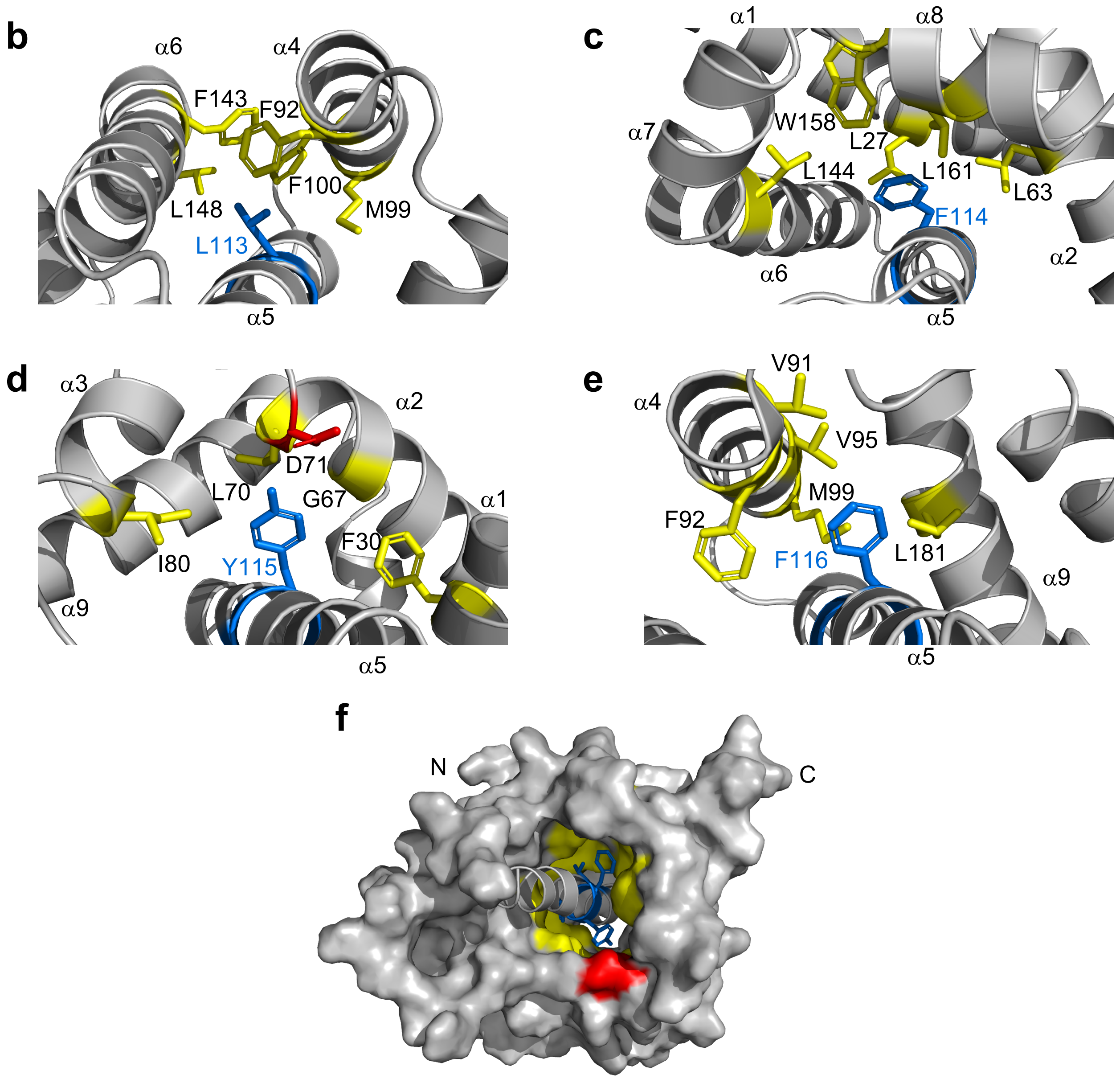

Supplementary Figure 1

Supplementary Figure 1. Network of residues engaged by BAX amino acids 113-116 of the α5 core. (a) Tabulated listing of BAX residues engaged by BAX α5 residues 113-116. (b-e) The network of amino acids that interact with residues 113-116 (blue) of the BAX α5 core through hydrophobic (yellow) or hydrogen bonding (red) contacts, as shown in ribbon diagrams for each of the four BAX α5 residues (b-e) and as an overall surface view (f).

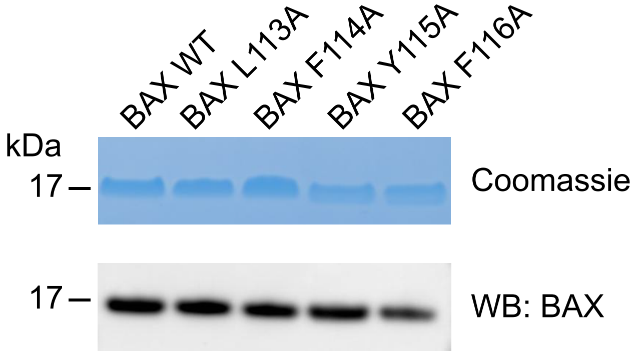

Supplementary Figure 2

Supplementary Figure 2. Production of full-length recombinant BAX and its single alanine mutants of the 113-116 nexus. Coomassie stain and western analysis of the indicated BAX proteins. The analysis was performed twice using independent preparations of proteins with similar results. Source data are provided as a Source Data file.

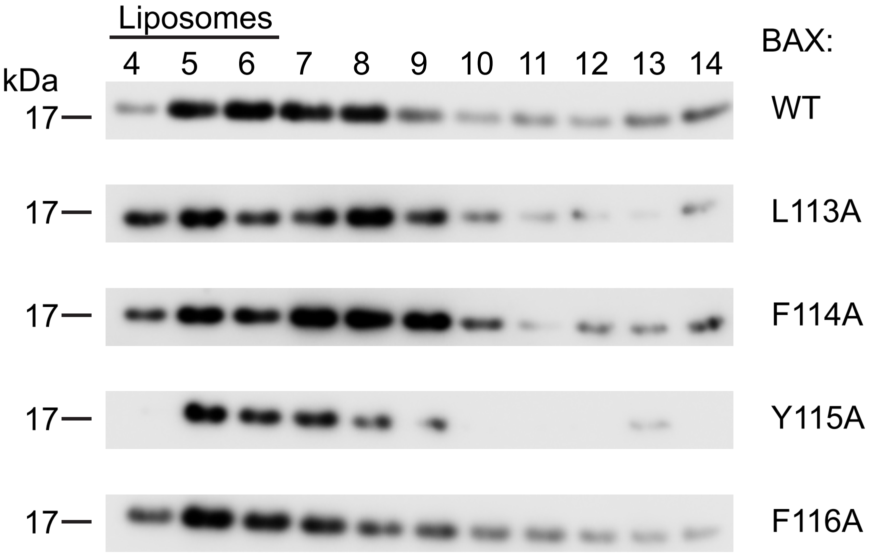

Supplementary Figure 3

Supplementary Figure 3. Liposomal translocation of tBID-triggered BAX mutants. tBID uniformly induces translocation of recombinant full-length BAX and its L113, F114, Y115, and F116 alanine mutants to the liposomal fractions, as demonstrated by BAX western analysis of fractions after 15 min incubation. The liposomal translocation assays were performed twice using independent preparations of liposomes and proteins with similar results. Source data are provided as a Source Data file.

a

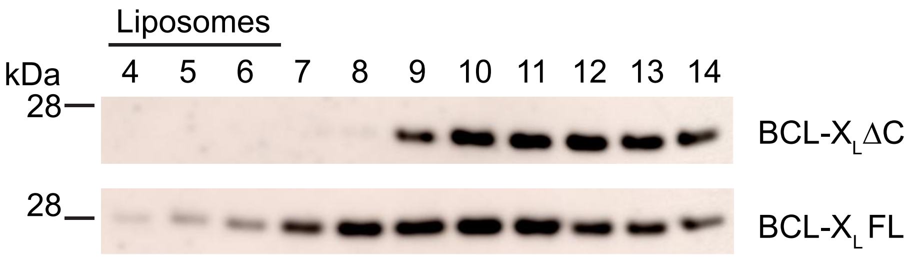

b

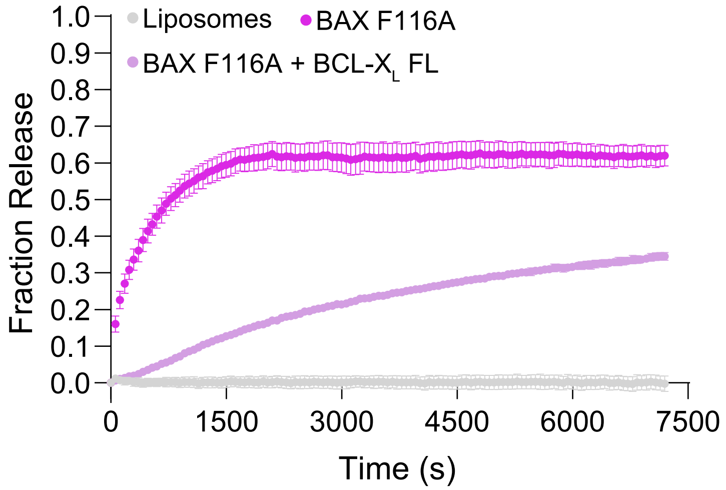

Supplementary Figure 4

Supplementary Figure 4. Liposomal partitioning of full-length BCL-X_L and its suppression of BAX F116A. (a) Whereas BCL-$X_L\Delta C$ remains fully in the aqueous phase, full-length BCL-X_L (BCL-X_L FL) independently partitions, in part, to liposomal membranes. The liposomal translocation assays were performed twice using independent preparations of liposomes and proteins with similar results. (b) Compared to BCL-$X_L\Delta C$ (Fig. 2o), BCL-X_L FL is more effective at suppressing BAX F116A-mediated liposomal release. Data are mean ± s.e.m. for liposomal release assays performed in technical quadruplicate and conducted twice using independent preparations of liposomes and proteins with similar results. Source data are provided as a Source Data file.

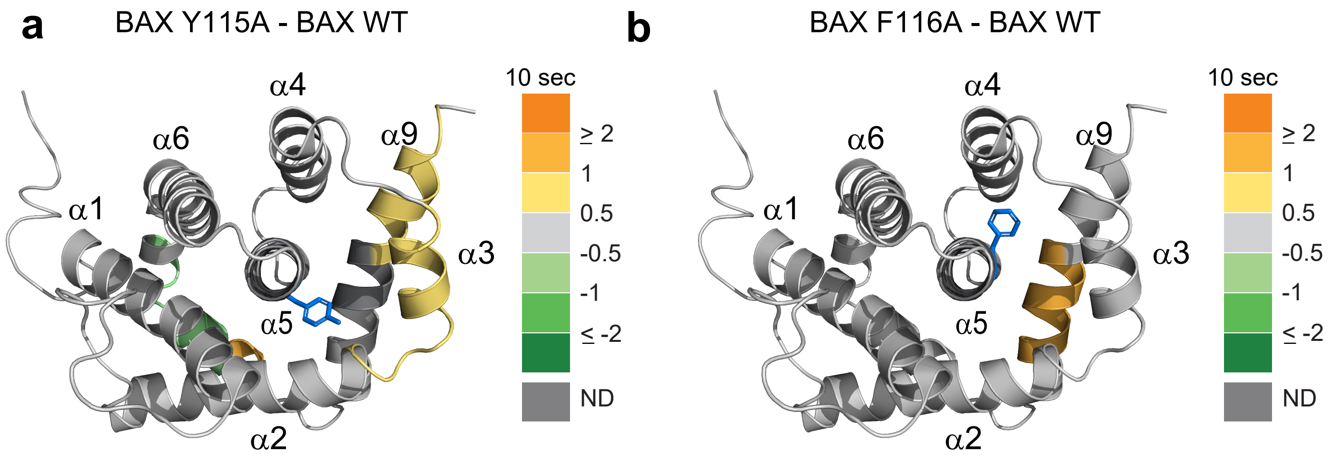

Supplementary Figure 5

Supplementary Figure 5. HDX MS analysis of BAX Y115A and BAX F116A reveals changes in α9 conformational dynamics. (a-b) Regions of relative deuterium deprotection (orange) and protection (green) above the 0.5 Da significance threshold at 10 sec of deuterium labeling are mapped onto the solution structure of BAX (PDB 1F16) for Y115A (a) and F116A (b), with the respective mutated residue colored in blue. HDX MS experiments were performed at least twice using independent preparations of BAX proteins. The HDX MS data used to create this figure can be found in Supplementary Data 2.

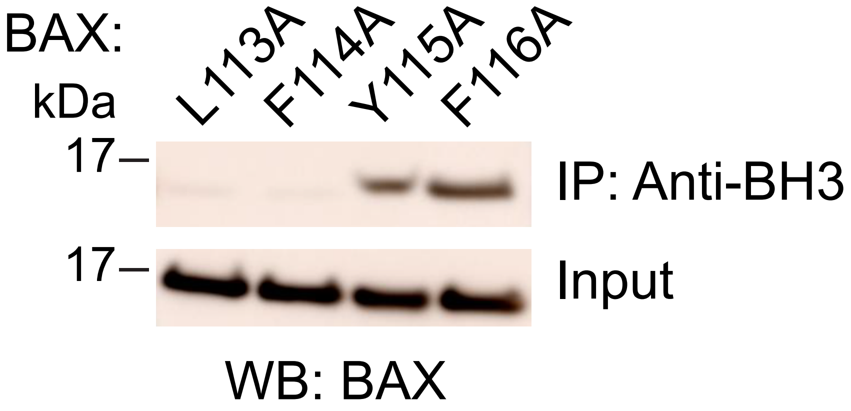
BAX:
kDa
L113A
F114A
Y115A
F116A
17
IP: Anti-BH3
17
Input
WB: BAX

Supplementary Figure 6. Comparative immunoprecipitation of BAX alanine mutants by a BAX BH3 antibody. The indicated single alanine BAX mutants were incubated with a BAX BH3 antibody, followed by immunoprecipitation, electrophoresis, and detection by BAX western analysis. BAX Y115A and BAX F116A, but not BAX L113A and F114A, were immunoprecipitated by the BAX BH3 antibody, consistent with conformational deprotection of the α2-α3 region in the two mutants, as detected by HDX MS (Fig. 4c-d). Immunoprecipitations were performed three times using independent preparations of proteins with similar results. Source data are provided as a Source Data file.

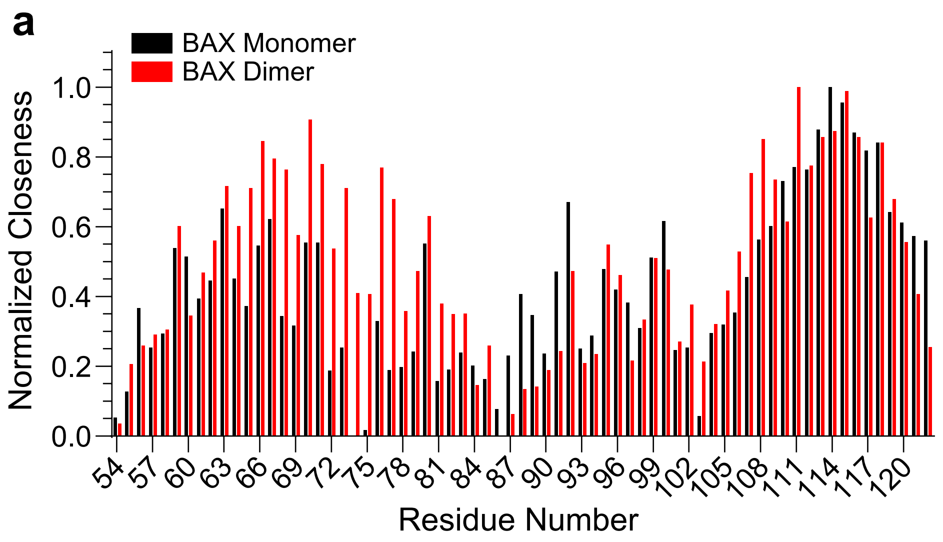

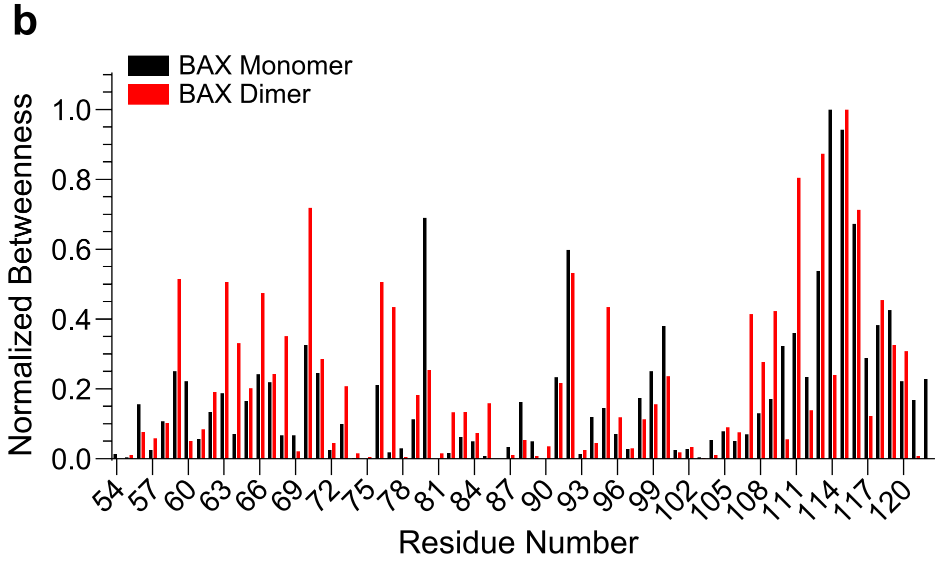

Supplementary Figure 7

Supplementary Figure 7. Comparative network analyses of monomeric and dimeric BAX. (a-b) Network analysis of BAX residues 54-122 (α2-α5) plotted as normalized closeness (a) and betweenness (b) for the monomer versus dimer. Source data are provided as a Source Data file.

a

Residue	Interacting Residue	Location
L113	92, 95, 96, 99,100	α4
	108, 109, 110, 111, 112, 114, 115, 116, 117, 118	α5
	63’	α2’
F114	109, 110, 111, 112, 113, 115, 116, 117, 118,	α5
	114’	α5’
Y115	70, 71	α2
	76, 77, 80	α3
	111, 112, 113, 114, 116, 117, 118, 119	α5
	107’, 108’, 111’	α5’
F116	80	α2
	92, 95	α3
	111, 112, 113, 114, 115, 117, 118, 119, 120	α5
	59’, 63’	α2’

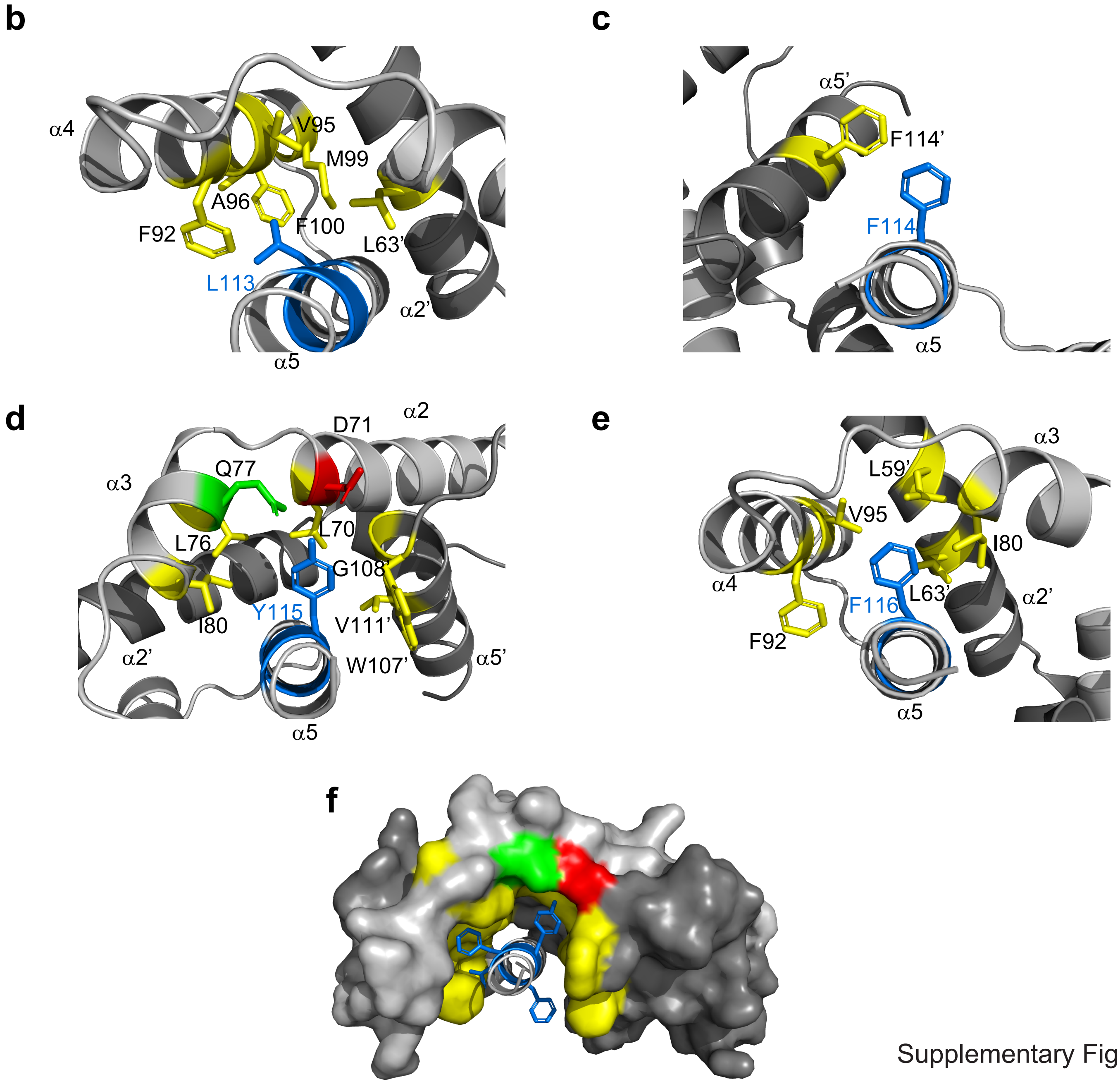

Supplementary Figure 8

Supplementary Figure 8. Network of residues engaged by BAX amino acids 113-116 of the BAX α2-α5 dimer. (a) Tabulated listing of BAX α2-α5 residues engaged by BAX α5 residues 113-116. (b-e) The network of amino acids that interact with residues 113-116 (blue) of the BAX α5 core through hydrophobic (yellow) or hydrogen bonding (red, green) contacts, as shown in ribbon diagrams for each of the four BAX α5 residues (b-e) and as an overall surface view (f).

Primer Name	Primer Sequence (5' to 3')	Application
BAX L113A_F	CTACTTTGCCAGCAAACTGGTGCTC	Mutagenic Primer of BAX
BAX L113A_R	AACGCGGCGACAACCCGGCCCCAGTT	Mutagenic Primer of BAX
BAX F114A_F	TGTCGCCCTTGCATACTTTGCCAGCAAACTG	Mutagenic Primer of BAX
BAX F114A_R	ACCCGGCCCCAGTTGAAG	Mutagenic Primer of BAX
BAX Y115A_F	CGCCCTTTTCGCATTTGCCAGCAAACTGGTGCTCAAG	Mutagenic Primer of BAX
BAX Y115A_R	ACAACCCGGCCCCAGTTG	Mutagenic Primer of BAX
BAX F116A_F	CCTTTTCTACGCAGCCAGCAAACTGGTGCTCAAGGCC	Mutagenic Primer of BAX
BAX F116A_R	GCGACAACCCGGCCCCAG	Mutagenic Primer of BAX
BAX L113A/F114A/Y115A/F116A_F	GCTGCAGCCAGCAAACTGGTGCTCAAGGC	Mutagenic Primer of BAX
BAX L113A/F114A/Y115A/F116A_R	AGCTGCGGCGACAACCCGGCCCCA	Mutagenic Primer of BAX
BAX L113A/F114A_F	GGTTGTCGCCGCTGCTTACTTTGCCAGCAAACTGG	Mutagenic Primer of BAX
BAX L113A/F114A_R	CGGCCCCAGTTGAAGTTG	Mutagenic Primer of BAX
BAX L113A/Y115A_F	GCTTTTGCCAGCAAACTGGTGCTCAAGGC	Mutagenic Primer of BAX
BAX L113A/Y115A_R	GAAAGCGGCGACAACCCGGCCCCA	Mutagenic Primer of BAX
BAX L113A/F116A_F	TACGCTGCCAGCAAACTGGTGCTCAAGGC	Mutagenic Primer of BAX
BAX L113A/F116A_R	GAAAGCGGCGACAACCCGGCCCCA	Mutagenic Primer of BAX
BAX F114A/Y115A_F	TGTCGCCCTTGCTGCTTTTGCCAGCAAACTGGTG	Mutagenic Primer of BAX
BAX F114A/Y115A_R	ACCCGGCCCCAGTTGAAG	Mutagenic Primer of BAX
BAX F114A/F116A_F	TACGCTGCCAGCAAACTGGTGCTCAAGGC	Mutagenic Primer of BAX
BAX F114A/F116A_R	AGCAAGGGCGACAACCCGGCCCCA	Mutagenic Primer of BAX
BAX Y115A/F116A_F	CGCCCTTTTCGCTGCTGCCAGCAAACTGGTGCTC	Mutagenic Primer of BAX
BAX Y115A/F116A_R	ACAACCCGGCCCCAGTTG	Mutagenic Primer of BAX
BAX L113A/F114A/Y115A_F	CGCAGCTGCTTTTGCCAGCAAACTGGTGC	Mutagenic Primer of BAX L113A/F114A/Y115A/F116A
BAX L113A/F114A/Y115A_R	GCGACAACCCGGCCCCAG	Mutagenic Primer of BAX L113A/F114A/Y115A/F116A
BAX L113A/F114A/F116A_F	CGCCGCAGCTTACGCAGCCAGCAAAC	Mutagenic Primer of BAX L113A/F114A/Y115A/F116A
BAX L113A/F114A/F116A_R	ACAACCCGGCCCCAGTTG	Mutagenic Primer of BAX L113A/F114A/Y115A/F116A
BAX L113A/Y115A/F116A_F	TGTCGCCGCATTTGCTGCAGCCAG	Mutagenic Primer of BAX L113A/F114A/Y115A/F116A
BAX L113A/Y115A/F116A_R	ACCCGGCCCCAGTTGAAG	Mutagenic Primer of BAX L113A/F114A/Y115A/F116A
BAX F114A/Y115A/F116A_F	GGTTGTCGCCCTTGCTGCTGCAG	Mutagenic Primer of BAX L113A/F114A/Y115A/F116A
BAX F114A/Y115A/F116A_R	CGGCCCCAGTTGAAGTTG	Mutagenic Primer of BAX L113A/F114A/Y115A/F116A
pTYB1_F	TGCTTTGCCAAGGGTACC	HiFi Assembly primer for pTYB1 vector
pTYB1_R	ATGTATATCTCCTTCTTAAAGTTAAACAAAATTATTTC	HiFi Assembly primer for pTYB1 vector
BCL-XL_F	TTTAAGAAGGAGATATACATATGTCTCAGAGCAACCGG	HiFi Assembly primer for BCL-XL
BCL-XL_R	TTGGTACCCTTGGCAAAGCATTTCCGACTGAAGAGTGAG	HiFi Assembly primer for BCL-XL
BCL-XL G138A_F	GGTAAACTGGGCTCGCATTGTGG	Mutagenic Primer of BCL-XL
BCL-XL G138A_R	CCATCCCGGAAGAGTTCAT	Mutagenic Primer of BCL-XL
BCL-XL ΔC_F	TGCTTTGCCAAGGGTACC	Mutagenic Primer of BCL-XL; same sequence as pTYB1_F
BCL-XL ΔC_R	GCGTTCCTGGCCCTTTCG	Mutagenic Primer of BCL-XL
pET28a_F	TAACAAAGCCCGAAAGGAAGCTGAGTTGGC	HiFi Assembly primer for pET28a vector
pET28a_R	ATGGCTGCCGCGCGGCAC	HiFi Assembly primer for pET28a vector
GFP_F	TGGTGCCGCGCGGCAGCCATATGAGCAAAGGTGAGGAGC	HiFi Assembly primer for GFP
GFP_R	TGGACGCATCGGAGCCAGTGATACCGGC	HiFi Assembly primer for GFP
BAX α2-α5_F	CACTGGCTCCGATGCGTCCACCAAGAAGC	HiFi Assembly primer for BAX α2-α5
BAX α2-α5_R	CTTCCTTTCGGGCTTTGTTACTTGGTGCTCAGGGCCTTG	HiFi Assembly primer for BAX α2-α5

Supplementary Table 1

Supplementary Table 1. Primers for generating recombinant BAX and BCL-X_L constructs.
