# Supplementary material for: The conformational stability of pro-apoptotic BAX is dictated by discrete residues of the protein core
Source: Nat Commun. 2021 Aug 13;12:4932. doi: 10.1038/s41467-021-25200-7 (PMC8363748; doi:10.1038/s41467-021-25200-7)
Supplement: Supplementary file 2 — Description of Additional Supplementary Files [file 41467_2021_25200_MOESM2_ESM.docx]

**Description of Additional Supplementary Files**

File Name: Supplementary Data 1

Description: HDX-MS experimental parameters

File Name: Supplementary Data 2

Description: HDX-MS data
