# Supplementary material for: The conformational stability of pro-apoptotic BAX is dictated by discrete residues of the protein core
Source: Nat Commun. 2021 Aug 13;12:4932. doi: 10.1038/s41467-021-25200-7 (PMC8363748; doi:10.1038/s41467-021-25200-7)
Supplement: Supplementary file 3 — Supplementary Data 1 [file 41467_2021_25200_MOESM3_ESM.pdf]

HDX Data Summary and Experimental Parameters

Data Set	BAX WT	BAX L113A	BAX F114A	BAX Y115A	BAX F116A	BAX α2-α5 WT	BAX α2-α5 F116A	BAX α2-α5 L113A/F114A/Y115A/F116A
**HDX reaction details**	Final D_2O concentration=94.7%, pH_{read}=6.80, 21 °C. See also footnote[a]							
**HDX time course**	10s, 1m, 10m							
**HDX undeuterated controls**	4	3				2	1	1
**Back-exchange**	30-35%[c]							
**Number of peptides**	67 followed; 92 identified					120 followed; 136 identified		
**Sequence coverage (%)**	92.7					91.5		
**Average peptide length; Redundancy**	11.3; 4.27					12.9; 5.19		
**Peptide filtering parameters**	0.25 products/amino acid; 2 consecutive products							
**Replicates**	See replication table below[c]							
**Repeatability[b]**	+/- 0.25 relative Da							
**Significant differences**	> 0.5 Da							

[a] **BAX constructs:** 18-fold dilution with labeling buffer [20 mM HEPES, 150 mM KCl, 1 mM $MgCl_2$, pD 7.2, 99.9% D_2O]. 1:1 dilution with quench buffer [0.8 M guanidinium chloride, 0.8% (v/v) formic acid, pH 2.0, H_2O]. **GFP-BAX α2-α5 constructs:** 18-fold dilution with labeling buffer [20 mM Tris, 150 mM KCl, pD 8.0, 99.9% D_2O]. 1:1 dilution with quench buffer [4 M guanidinium chloride, 200mM potassium phosphate, 0.72 M TCEP, pH 2.34, H_2O].

[b] No statistical tests were applied to the HDX MS measurements. Rather, based on measurements of mean methodological error (+/- 0.14 Da; (Houde D, Berkowitz SA, Engen JR. *J. Pharm. Sci.* 2011;100(6):2071-2086.) we chose a value (+/- 0.25 Da) well above that as the threshold for calling differences in relative deuterium incorporation measurements meaningful. See also explanations of this methodology in (Engen JR, Wales TE. *Annu. Rev. Anal. Chem.* 2015;8:127-148.).

[c] **Replication**

Time point	BAX WT	BAX L113A	BAX F114A	BAX Y115A	BAX F116A	BAX α2-α5 WT	BAX α2-α5 F116A	BAX α2-α5 L113A/F114A/Y115A/F116A
	Number of replicates							
**undeuterated controls**	4	3	3	3	3	2	1	1
**10 sec**	3	2	2	2	2	2	2	2
**1 min**	3	2	2	3	2	2	2	2
**10 min**	3	2	2	3	2	2	2	2
