# Supplementary material for: The conformational stability of pro-apoptotic BAX is dictated by discrete residues of the protein core
Source: Nat Commun. 2021 Aug 13;12:4932. doi: 10.1038/s41467-021-25200-7 (PMC8363748; doi:10.1038/s41467-021-25200-7)
Supplement: Supplementary file 5 — Reporting Summary [file 41467_2021_25200_MOESM5_ESM.pdf]

nature research

Corresponding author(s): Loren D. Walensky

Last updated by author(s): July 17, 2021

Reporting Summary

Nature Research wishes to improve the reproducibility of the work that we publish. This form provides structure for consistency and transparency in reporting. For further information on Nature Research policies, see our Editorial Policies and the Editorial Policy Checklist.

Statistics

For all statistical analyses, confirm that the following items are present in the figure legend, table legend, main text, or Methods section.

n/a	Confirmed	
☐	☒	The exact sample size (*n*) for each experimental group/condition, given as a discrete number and unit of measurement
☐	☒	A statement on whether measurements were taken from distinct samples or whether the same sample was measured repeatedly
☒	☐	The statistical test(s) used AND whether they are one- or two-sided<br>*Only common tests should be described solely by name; describe more complex techniques in the Methods section.*
☒	☐	A description of all covariates tested
☒	☐	A description of any assumptions or corrections, such as tests of normality and adjustment for multiple comparisons
☐	☒	A full description of the statistical parameters including central tendency (e.g. means) or other basic estimates (e.g. regression coefficient) AND variation (e.g. standard deviation) or associated estimates of uncertainty (e.g. confidence intervals)
☒	☐	For null hypothesis testing, the test statistic (e.g. *F*, *t*, *r*) with confidence intervals, effect sizes, degrees of freedom and *P* value noted<br>*Give P values as exact values whenever suitable.*
☒	☐	For Bayesian analysis, information on the choice of priors and Markov chain Monte Carlo settings
☒	☐	For hierarchical and complex designs, identification of the appropriate level for tests and full reporting of outcomes
☒	☐	Estimates of effect sizes (e.g. Cohen's *d*, Pearson's *r*), indicating how they were calculated

Our web collection on statistics for biologists contains articles on many of the points above.

Software and code

Policy information about availability of computer code

Data collection	Network centrality data was generated by the NAPS server (https://bioinf.iiit.ac.in/NAPS/). Fluorescence and absorbance data were collected using SoftMax Pro 7.0.2 and i-control 1.11 software. HDX-MS data were processed and analyzed using PLGS 3.0.1 and DynamX 3.0.
Data analysis	Data were analyzed using Microsoft Excel 2016 and Prism 8 (Graphpad) software.

For manuscripts utilizing custom algorithms or software that are central to the research but not yet described in published literature, software must be made available to editors and reviewers. We strongly encourage code deposition in a community repository (e.g. GitHub). See the Nature Research guidelines for submitting code & software for further information.

Data

Policy information about availability of data

All manuscripts must include a data availability statement. This statement should provide the following information, where applicable:

- Accession codes, unique identifiers, or web links for publicly available datasets
- A list of figures that have associated raw data
- A description of any restrictions on data availability

All data generated or analyzed for this study are included in this manuscript and its supplementary information. HDX-MS have been deposited to the ProteomeXchange Consortium via the PRIDE partner repository with the dataset identifier PXD024479 [https://www.ebi.ac.uk/pride/archive/projects/ PXD024479]. Structures corresponding to PDB 1F16 [http://doi.org/10.2210/pdb1F16/pdb] and 4BDU [http://doi.org/10.2210/pdb4BDU/pdb] were used in this study. Source data are provided with this paper.

April 2020

Field-specific reporting

Please select the one below that is the best fit for your research. If you are not sure, read the appropriate sections before making your selection.

☒ Life sciences ☐ Behavioural & social sciences ☐ Ecological, evolutionary & environmental sciences

For a reference copy of the document with all sections, see nature.com/documents/nr-reporting-summary-flat.pdf

Life sciences study design

All studies must disclose on these points even when the disclosure is negative.

Sample size	All experiments were performed in n=3 to 4 technical replicates to ensure an accurate analysis of the average and standard deviation of a measurement, and then repeated in at least biological duplicate using independent experimental preparations (e.g. protein, mitochondria, cells). Sample sizes were selected based on standard practices for these assays in the cell death field, as referenced in the manuscript, and provide reproducible results as demonstrated by plots of replicate data points, mean, and s.d. or s.e.m. Sample sizes for each experiment are reported in the figure legends.
Data exclusions	No data were excluded during analysis.
Replication	All experiments were reproducibly performed in at least biological duplicate.
Randomization	No experimental grouping or randomization was used in this study.
Blinding	Blinding is not applicable for the biochemical, cellular, and HXMS experiments because data acquisition is performed by instrumentation and analytical software, with data detection and collection not influenced by the operator.

Reporting for specific materials, systems and methods

We require information from authors about some types of materials, experimental systems and methods used in many studies. Here, indicate whether each material, system or method listed is relevant to your study. If you are not sure if a list item applies to your research, read the appropriate section before selecting a response.

Materials & experimental systems

n/a	Involved in the study
☐	☒ Antibodies
☐	☒ Eukaryotic cell lines
☒	☐ Palaeontology and archaeology
☐	☒ Animals and other organisms
☒	☐ Human research participants
☒	☐ Clinical data
☒	☐ Dual use research of concern

Methods

n/a	Involved in the study
☒	☐ ChIP-seq
☒	☐ Flow cytometry
☒	☐ MRI-based neuroimaging

Antibodies

Antibodies used	anti-BAX (2D2, Santa Cruz Biotechnology, Cat# sc-20067); anti-BCL-XL (2H12, Abcam, Cat# ab270253); anti-VDAC1 (Abcam, Cat# ab186321); anti-LDH (Abcam, Cat# ab47010); anti-Actin (Cell Signaling Technology, Cat# 5125); anti-BAX BH3 (Abcepta, Cat# AP1302a)
Validation	All antibodies were validated by the manufacturers to interact with the proteins used in this study.<br>Anti-BAX 2D2: https://www.scbt.com/p/bax-antibody-2d2; reacts with mouse, rat, and human origin. Applications: WB, IP, IF, IHC, F. Anti-BCL-XL 2h12: https://www.abcam.com/bcl-xl-antibody-2h12-ab270253.html; reacts with human. Applications: WB, IHC. Anti-VDAC1: https://www.abcam.com/vdac1porin-antibody-n152b23-ab186321.html; reacts with rodent, human. Applications: WB, ICC, IF. Anti-LDH: https://www.abcam.com/lactate-dehydrogenase-antibody-ab47010.html; reacts with human (our data demonstrates mouse reactivity). Applications: WB, IP, ICC/IF/IHC. Anti-Actin: https://www.cellsignal.com/products/antibody-conjugates/b-actin-13e5-rabbit-mab-hrp-conjugate/5125; reacts with human, mouse, rabbit, mokey, bovine, and pig. Applications: WB. Anti-BAX BH3: https://www.abcepta.com/products/AP1302a-Bax-Antibody-BH3-Domain-Specific; reacts with human and mouse. Applications: WB, F, E.

Eukaryotic cell lines

Policy information about cell lines

Cell line source(s)	BAX-/- BAK-/- MEFs were purchased from ATCC (CRL-2913); GPG-293 cells were provided by A. Cantor (Boston Children's Hospital)
Authentication	Authentication performed by supplier. ATCC uses morphology, karyotyping, and PCR-based methods to confirm the identify of mouse cell lines and to rule out intra- and interspecies contamination. GPG-293 cells were used as a reagent for retroviral packaging and production only, which was successfully achieved for MEF reconstitution studies.
Mycoplasma contamination	Cell lines tested negative for mycoplasma (MycoAlert, Lonza Biologics)
Commonly misidentified lines (See ICLAC register)	No commonly misidentified eukaryotic cell lines were used in this manuscript.

Animals and other organisms

Policy information about studies involving animals; ARRIVE guidelines recommended for reporting animal research

Laboratory animals	Mitochondria were isolated from the livers of Alb-creposBaxf/fBak-/- mice.
Wild animals	The study did not involve wild animals.
Field-collected samples	The study did not involve field-collected samples.
Ethics oversight	Liver harvest from Alb-creposBaxf/fBak-/- mice for mitochondrial isolation was performed in accordance with the guidelines and regulations set forth by the Institutional Animal Care and Use Committee of the Dana-Farber Cancer Institute and in compliance with approved study protocol #06-004. Animals were housed in microisolater cages at an ambient temperature of 68-79°F and humidity of 30-70%, with 12-hour light/dark cycles.

Note that full information on the approval of the study protocol must also be provided in the manuscript.
